# Engagement of people with lived experience in the design and development of digital mental health interventions: A scoping review of engagement characteristics and impacts

**DOI:** 10.1016/j.invent.2026.100914

**Published:** 2026-01-29

**Authors:** Alana Fisher, Noni Jervis, Madelyne Bisby, Milena Gandy, Andreea I. Heriseanu, Taylor Hathway, Atria Rezwan, Nickolai Titov, Blake Dear

**Affiliations:** a eCentreClinic, School of Psychological Sciences, Faculty of Medicine, Health, and Human Sciences, Macquarie University, NSW, Australia; b MindSpot, MQ Health, Macquarie University, NSW, Australia

**Keywords:** Scoping review, Lived experience, Digital mental health interventions, Intervention development, Benefits, Challenges, Outcomes

## Abstract

**Background:**

Digital mental health interventions (DMHIs) aim to increase access to mental healthcare for people who would otherwise not access it. Accordingly, the design and development of DMHIs may particularly benefit from engaging people with lived experience (PwLE).

**Methods:**

A scoping review involving systematic database searches identified and synthesised original research reporting PwLE engagement in the design and development of DMHIs (published January 2000– April 2024). Articles were independently title/abstract screened by two authors, and full-text screened by one author. Included article data were extracted, independently checked, and descriptively synthesised.

**Results:**

Twenty-nine studies were included, published 2012–2024, in high-income countries. Engagement was mostly via ‘consultation’ level activities (e.g., focus groups), followed by ‘involvement’ or ‘collaboration’. In almost half of studies, engagement spanned multiple engagement levels across the different development stages. Reported impacts included changes to content, design, and delivery (e.g., revised language). Authors credited engagement with improved DMHI relevance, acceptability, and inclusivity, while noted challenges include limited diversity among engaged PwLE and resource demands. For reported DMHI outcomes, studies reported positive findings related to use, and attitudes towards using DMHIs. However, reported findings were more mixed for mental health outcomes (e.g., symptom improvement). Additionally, it was not possible to directly link outcomes to PwLE engagement.

**Conclusions:**

Review findings highlight the increasing but predominantly consultative engagement of PwLE in developing DMHIs. Future research directions include more transparent and consistent reporting of engagement, deliberative decision-making around engagement levels/types, and more rigorous evaluation of engagement to investigate its association with DMHI outcomes.

## Highlights

• Design of DMHIs may particularly benefit from engaging people with lived experience.
• Scoping review synthesised 29 studies reporting PwLE engagement in DMHI development.
• Engagement was mostly consultative; changes spanned DMHI design, content, delivery.
• Benefits were commonly reported, but direct links to DMHI outcomes remain unclear.
• More detailed reporting and rigorous evaluation of engagement in DMHI research needed.

## 1 Introduction

Recent years have seen a shift away from mental health research led by researchers towards approaches that engage or meaningfully involve people with lived (or living) experience of a mental health condition or service use. Lived experience may refer to a person's first-hand personal experience of a mental health condition or service use (i.e., as a consumer), or as the family member, loved one, kin, or support person (Dray et al., 2024). Within these engagement approaches, people with lived experience (PwLE) are sought not as research participants but as consultants, advisors, collaborators, and/or co- or lead investigators to provide the expert knowledge and skills they have gained by way of their lived experience, as opposed to professional training and/or qualifications (Dray et al., 2024). These engagement approaches broadly aim to ensure that the perspectives, preferences, and needs of PwLE guide the decisions made throughout the research lifecycle, from research planning and priority setting to execution and translation.

By harnessing a complementary form of expertise alongside professional expertise, engaging PwLE in health research is said to facilitate research that better addresses consumer needs, is translatable into policy and practice, and in turn leads to improved patient outcomes (Cochrane Board, 2017). In addition, prominent health research funding bodies both in Australia (e.g., National Health and Medical Research Council, [NHMRC] (NHMRC, 2016)) and in other high income western countries overseas (e.g., the UK's National Institute for Health and Care Research [NIHR] (National Institute for Health and Care Research, 2024), the US's Patient-Centered Outcomes Research Institute [PCORI] (Patient-Centered Outcomes Research Institute, 2024)) now require and give substantial weighting to the engagement of PwLE throughout grant application assessment.

Research on the design, development and delivery of digital mental health interventions (DMHIs) may be one area which particularly benefits from engaging PwLE. These DMHIs are psychoeducation, psycho-support, or skill-based interventions delivered via an internet-connected device (e.g., smartphone, laptop/desktop computer, or tablet), which are designed to help manage, treat, or prevent the development of mental health symptoms. They include a broad range of tools and programs (e.g., apps, websites, games, virtual reality) in a self-guided/unguided (i.e., self-help, self-management) or guided format (e.g., with support from a health professional or non-clinical personnel) (Borghouts et al., 2021). As one of the key purported advantages of DMHIs is in increasing access to evidence-based care for underserved groups who cannot or choose not to access traditional mental health treatments (Graham et al., 2020), the design and development of these interventions may benefit from engaging PwLE whose perspectives may elucidate, among other issues, barriers and solutions to accessing, using, and gaining benefit from DMHIs.

To date, there are no known reviews specifically focussing on the engagement of PwLE in the design and development of DMHIs in research settings. Broader reviews of mental health research designs, intervention design, face-to-face care and youth have revealed some consistent trends in lived experience engagement. For example, lived experience engagement in intervention design is typically limited to consultation rather than co-design (Barker et al., 2024; Bevan Jones et al., 2020; Orlowski et al., 2015; Slay and Stephens, 2013; Veldmeijer et al., 2023), despite reported benefits (e.g., improved relevance and engagement) (Bevan Jones et al., 2020; Sheikhan et al., 2023), with persistent barriers (e.g., sustaining involvement, resource constraints, tokenism) and methodological gaps in how engagement is defined, implemented, and evaluated (Bevan Jones et al., 2020; Orlowski et al., 2015; Veldmeijer et al., 2023; Sheikhan et al., 2023; Hawke et al., 2023) (Bevan Jones et al., 2020; Orlowski et al., 2015; Hawke et al., 2023). .However, it remains unknown whether these trends extend to DMHI research in adults. A synthesis of this research would allow researchers to make use of what is already known to guide their practice of engaging PwLE, inform efforts to build the evidence base, and address current gaps.

### 1.1 Review questions

With reference to published original research, the following questions were posed in the current review:

‐ **Question 1:** What is the engagement or active involvement of PwLE in the design/development of DMHIs over time and by country/region?
‐ **Question 2:** What type/s of engagement with PwLE have been described in developing DMHIs?
‐ **Question 3:** What type/s of DMHIs have been developed by engaging PwLE? (e.g., format, modality, target population or condition)
‐ **Question 4:** What reported change/s have been made to the design, content, format, or delivery of DMHIs developed with engagement of PwLE?
‐ **Question 5:** What are the reported benefits or positive impacts of engaging PwLE in digital mental health research?
‐ **Question 6:** What are the reported challenges, costs or negative impacts of engaging PwLE in digital mental health research?
‐ **Question 7:** What are the reported outcomes of DMHIs developed with engagement of PwLE, in terms of:
  a. **Use:** initial uptake and ongoing engagement
  b. **End-user attitudes towards using:** acceptability, satisfaction
  c. **Efficacy or effectiveness:** improvements in mental health symptoms.

## 2 Methods

### 2.1 Study design

We undertook a scoping review for several reasons, including that we expected the available evidence to be heterogenous in terms of study design, methods and assessed outcomes (Peters et al., 2020). As a scoping review, an assessment of the quality of articles (and resulting recommendations for practice) was not performed. Guided by Arksey and O’Malley's (Arksey and O'Malley, 2005) framework and subsequent recommendations (Colquhoun et al., 2014; Levac et al., 2010; Munn et al., 2018), the review team developed a systematic search strategy protocol based on the PI(*E*)COS (Population, Intervention/Exposure, Comparator, Outcome/s, Setting) framework (see Table 1).

**Table 1:** Summary of PICOS framework used to guide searches^a^.

Population	People aged 16 years and older with lived/living experience of mental health distress and/or of supporting someone with mental health distress, who were actively engaged in the design or development of a digital mental health intervention.
Intervention	Digital interventions delivered at least partly online that are designed to treat, improve, manage, or prevent mental health symptoms or psychological distress, include both psychoeducation and psychosocial or psychological support, and are intended for people aged 16 years and older.
Comparison	With or without a comparison or control group.
Outcomes	Reported outcomes related to intervention or treatment development, intervention use or acceptability, effectiveness or efficacy for mental health outcomes, and/or other benefits, challenges, or costs associated with engaging people with lived experience.
Setting	Conducted in research and/or routine care settings, including universities, research institutes, clinics, or service development and quality improvement contexts.

a For full description of eligibility criteria see Supplementary Table 2.

### 2.2 Search strategy

Articles were identified through systematic searches of four key electronic databases (MEDLINE, EMBASE, PsycINFO, and Scopus). Search results were limited to studies including human participants, published in English and between 1 January 2000, and 18 April 2024.

Initial keyword search strategies were guided by recent reviews on engaging PwLE of mental health and alcohol/other drugs use concerns in research (Sheikhan et al., 2023) and factors influencing use of DMHIs (Borghouts et al., 2021; Forbes et al., 2023). These strategies were then refined in discussions with the review team, a specialist academic librarian, and a lived experience researcher (co-author; AR). Including a lived experience perspective was important to ensure that the research questions (and therefore, resulting findings) were seen to be relevant and potentially impactful for the broader non-academic community.

Supplementary Table 1 provides an example search query used for MEDLINE organised using terms related to three conceptual domains:

1) Lived experience in research (lines 1 to 4)
2) Mental health distress/diagnosis (lines 6 to 7)
3) Digital intervention (lines 9 to 12)

### 2.3 Data screening

All initial data screening was completed using Covidence (https://www.covidence.org/). After removing duplicates, titles and abstracts were independently screened by two reviewers (two of AF, NJ, MB, MG, AH) for potential inclusion using the specified eligibility criteria (see Supplementary Table 2). After title-abstract screening a random selection of 100 articles, the reviewers met to ensure that they were interpreting the eligibility criteria in the same way. Article full texts were then screened by one reviewer (AF).

### 2.4 Data extraction

A data extraction proforma was drafted in Excel by the first author (AF). To ensure common understanding and use, the proforma was piloted by AF and a second reviewer (NJ) using a random sample of the included full-text articles (~10.0%). Following piloting and refinements to the proforma, all data was extracted by NJ. A second reviewer (TH) then independently checked a random 25.0% sample of articles.

Throughout the data screening and data extraction process, discrepancies and areas needing clarification were resolved in discussion with the review team. See Supplementary Table 3 for the final set of extracted article characteristics.

### 2.5 Data analysis and synthesis

All extracted article characteristics (see Supplementary Table 3) were descriptively analysed and synthesised by the first author (AF). Determined level/s of engagement were according to the “Levels of Patient and Researcher Engagement in Health Research” framework (Manafò et al., 2018). This multilevel framework sets out a **continuum that describes the degree to which patients (and other lived-experience stakeholders) are involved in health research decision-making**, ranging from minimal involvement to shared leadership and control. Adapted from the **International Association for Public Participation (IAP2)’s Spectrum of Public Participation (****International Association for Public Participation,** 2018**), it is** used to clarify roles, expectations, and power-sharing in research. This framework was chosen because it related to health research specifically and clearly defined each level of engagement (learn/inform, participate, consult, involve, collaborate, lead/support) in terms of the respective roles and responsibilities of PwLE, and researchers, and examples of activities. Moreover, there is a lack of consistency in how terms such as “codesign” and “coproduction” are used in the literature (Slattery et al., 2020), such that authors may use them to denote more limited levels of engagement (e.g., using “codesign” instead of “consultation”). In determining the level/s of engagement, AF relied on the nature of engagement and types of activities reported, along with the extent to which engagement had an influence on reported changes or decisions made. In instances where the level/s of engagement were unclear or appeared to sit on a spectrum between two levels, this was noted. Where possible, preliminary links were described between engagement characteristics (i.e., type/s and level/s) and the reported impacts or outcomes.

## 3 Results

**Fig. 1:**
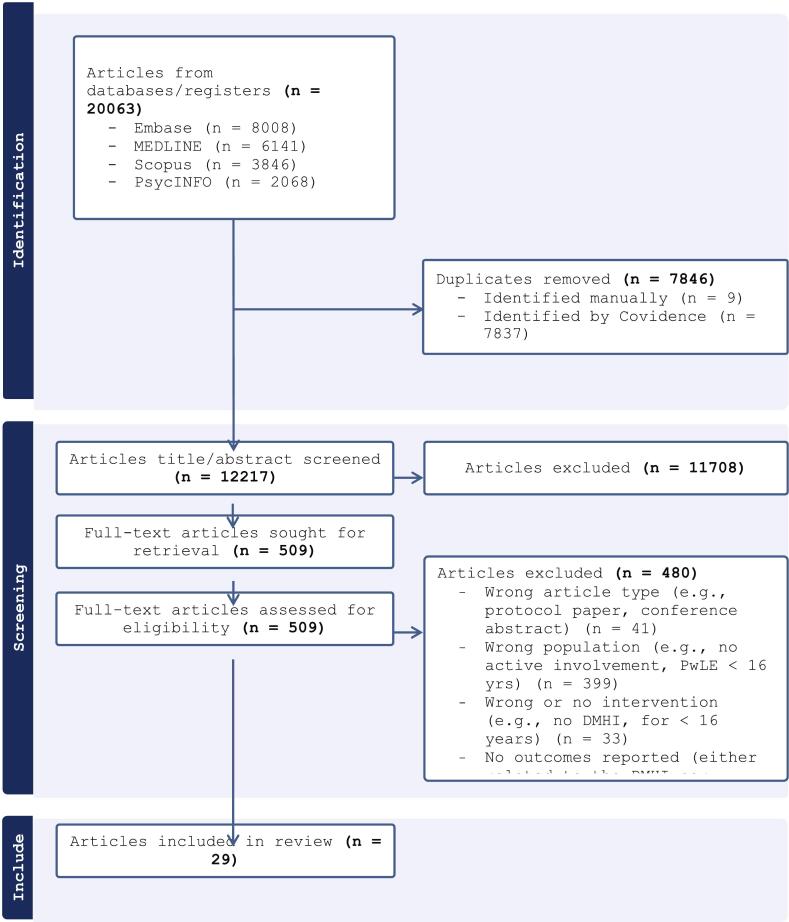
PRISMA flowchart.

As can be seen in the PRISMA flowchart (Fig. 1), database searches returned a total of 20,063 articles, 7846 of which were removed as duplicates. After title/abstract screening 12,217 articles, 11708 were excluded leaving 509 full-text articles for retrieval and review. Based on full-text review, 480 articles were excluded leaving a final sample of 29 articles of various study designs for inclusion (i.e., 12 x qualitative, 7 x mixed-method, 6 x randomised controlled trials [RCTs], 3 x single-arm/open trial, 1 x quantitative only).

The main reason for excluding articles at the full-text review stage was because the studies did not report any engagement of PwLE either at all (i.e., limited to study participation), or in design or development of an intervention. For the full data extraction of the included studies see Supplementary Tables 4 and 5.

**Question 1:** year-by-year increases and by geographical concentration of engagement of people lived experience.

**Fig. 2:**
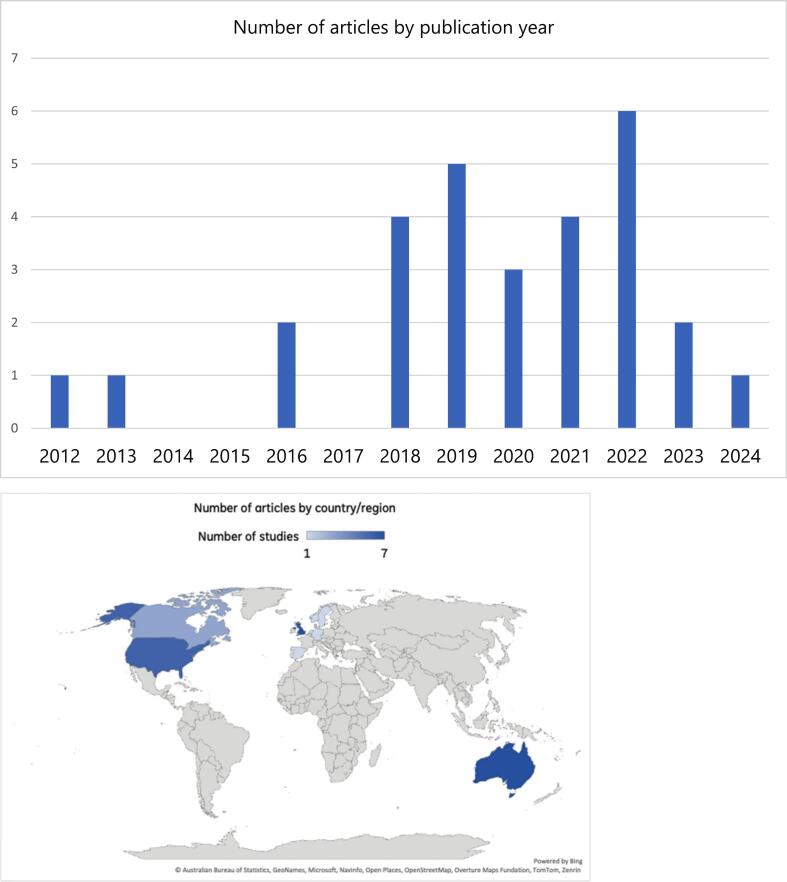
Number of articles by year of publication (top) and by country/region (bottom).

As seen in Fig. 2 (top), all included studies were published after 2012, with the largest number in 2019 (*n* = 5; 17.2%) and 2022 (*n* = 6; 20.7%). The one included study in 2024 likely reflects the end-date for searches (April 2024).

As seen in Fig. 2 (bottom), included studies were mostly conducted in Australia (*n* = 7; 24.1%), the United Kingdom (UK; n = 7; 24.1%), and the United States (US; n = 6; 20.1%), followed by Canada (*n* = 3; 10.3%), Central and Northern Europe (i.e., Denmark, *n* = 2; 6.9%; Germany, Netherlands, Norway, Spain, Sweden, *n* = 1 or 3.4% each).

**Question 2:** Type/s of engagement (level/s and activities).

Included studies employed a range of engagement levels and activities, as summarised and described in Table 2. Across the included studies, “consult” appeared to be the most common level of engagement (*n* = 26; 89.7%), followed by “involve” (*n* = 13; 44.8%), and “collaborate” (*n* = 4; 13.8%). None of the included studies appeared to be lived experience led. In this way, engagement tended to be one-off or intermittent, as opposed to ongoing or continuous throughout, and power and influence over development decisions rested mainly with (non-PwLE) researchers (Table 2).

**Table 2:** Summary of engagement with people with lived experience across the included articles.

	Level/s of engagement			
	Consult	Involve	Collaborate	Lead/support
	*Providing (targeted) feedback and advice on specific aspects of intervention design, development and/or delivery.*	*Working directly with the research team throughout the intervention design, development and/or delivery process*	*Partnering equally with the researchers as project team members working across all aspects of intervention design, development, and delivery.*	*Making decisions and leading activities for intervention design, development, and delivery*
Engagement activity examples	*Needs assessment or feedback surveys, qualitative interviews, focus groups, “think aloud” feedback sessions, review of intervention materials and/or testing (prototype, pilot, beta, usability), members of* ad hoc *working groups, or expert panels.*	*Members of standing working groups, iterative workshop series, project reference groups or advisory boards or committees.*	*Co-investigators/co-researchers and research partners, members of research/project steering committees.*	*Principal/lead investigators, consumer, carer or community-based steering committees.*
Decision-making power and influence (held by people with lived experience relative to researchers)	*None or limited*	*Limited or shared (at discrete points)*	*Shared (embedded throughout)*	*Prioritising people with lived experience.*
Nature of engagement	*One-off with/without follow-up*	*Regular time points*	*Ongoing*	*Integral and consistently sustained throughout*
Author name, year | intervention				
Abraham, 2018 | CALM (adapted)	+			
Batchelor, 2022 | COPe-support	+	+		
Behr, 2024 | TONI	+			
Ben-Zeev, 2013 | FOCUS	+			
Bucci, 2019 | Actissist	+	+		
Callan, 2021 | CBT Mobile Work	+			
Danaher, 2012 | MomMoodBooster	+			
Flobak, 2021 | My ADHD		+		
Geerling, 2022 | Wellbeing Bipolar	+			
Geraghty, 2016 | Health Paths Through Stress	+			
Guala, 2023 | Maze Out	+	*	*	
Hidalgo-Mazzei, 2016 | SIMPLe	+			
Honary, 2018 | REACT (web-based)	+	+		
Hughes-Barton, 2023 | i can act now	+	+		
Lal, 2020 | Horyzons	+	+		
Lederman, 2019 | Meridian	+			
Lehavot, 2021 | DESTRESS-WV	+			
MacKinnon, 2022 | BEAM	*	*		
McClelland & Fitzgerald, 2018 | (Mobile app – unnamed)	+			
Midgley, 2021 | D:OTS	+			
Milgrom, 2020 | MumMoodBooster	+			
Ospina-Pinillos, 2019 | Mental Health eClinic (MHeC-S)	+	+		
Patterson, 2022 | Tranquillity	*	*		
Reupert, 2020 | mi.spot	+	+		
Sin, 2019 | COPe-support	+		+	
Terp, 2018 | MindFrame	*	*		
Torok, 2022 | LifeBuoy	+			
Whiteside, 2019 | NowMattersNow.org			+	
Wiberg, 2022 | NARA		*	*	

Notes

+ = Level/s of engagement based on the nature/types of reported activities and extent of influence on decision-making.

* = Difficult to determine level of engagement, appears to sit on a spectrum between two levels based on the nature/types of reported activities and extent of influence on decision-making.

As many of the included studies were multi-stage and included different types of engagement activities, almost half (*n* = 13; 44.8%) engaged PwLE at more than one level (Table 1). Here the different levels mapped onto different activities and/or stages of intervention development. Across these studies, levels were most commonly “consult” and “involve” (*n* = 10; 34.5%). For five of the included studies (17.2%), it was unclear which level of engagement was most fitting, with reported activities appearing to sit between two adjacent levels, namely “consult” and “involve” (*n* = 3; 10.3%) or “involve” and “collaborate” (*n* = 2; 7.0%).

In terms of specific activities engaging PwLE, studies variously described: focus groups (*n* = 15; 51.7%) (one-off or multiple), (semi-structured) interviews (*n* = 11; 37.9%), prototype or usability testing (incl. “think aloud”) (*n* = 10; 34.5%), workshops integrating co-design or iterative design elements (*n* = 8; 27.6%) (one-off or multiple), surveys/questionnaires (*n* = 6; 20.7%), advisory groups or expert panels (n = 6), asynchronous written feedback methods (e.g., via email or online forms), observations with “think aloud” (independent of usability testing described above) (*n* = 3; 10.3%), community forums/discussions (n = 3; 10.3%), and co-production of videos/multimedia (*n* = 2; 7.0%). For full details of engagement activities see Supplementary Table 4.

**Question 3:** Characteristics of DMHIs and the PwLE/other engaged stakeholders.

**Fig. 3:**
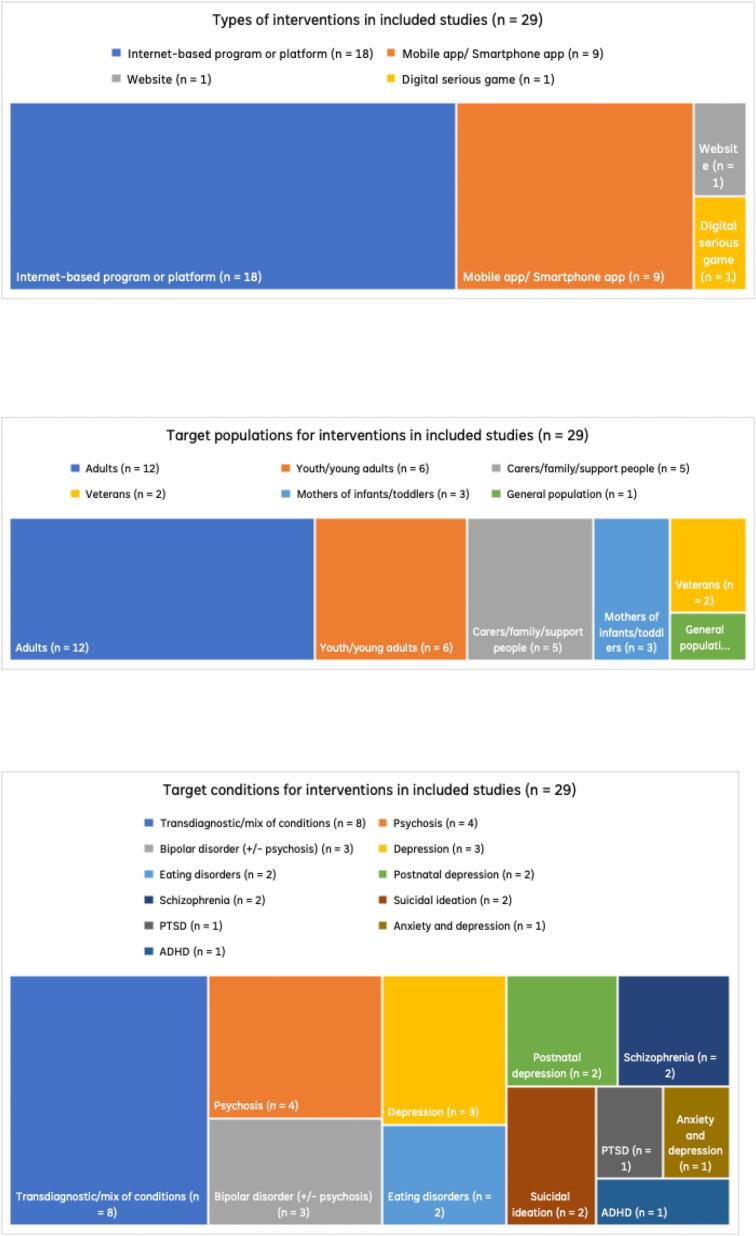
Types of interventions (top), target populations (middle), and target conditions of interventions (bottom) in included studies (n = 29).

As seen in Fig. 3 (top), the majority of studies (*n* = 18; 62.1%) included DMHIs which were variously described as online, internet- or web-based psychological treatment programs or platforms. These tended to be structured, multicomponent interventions that comprised psychoeducation and/or skill-based “modules” to complete, either in a guided (e.g., with the support of clinician or ‘coach’) or self-guided way (e.g., self-management). Although not all DMHIs were based on a specific theoretical approach, Cognitive Behavioural Therapy (CBT) was the most common, either alone or in combination with other approaches (*n* = 10; 34.5%).

As seen in Fig. 3 (middle), DMHIs were designed most commonly for adults in general (*n* = 12; 41.4%) or at particular life stages (e.g., youth, veterans, postpartum; *n* = 11; 37.9%). Most DMHIs were for consumers, with five of the 29 (17.2%) for carers, family or support people. DMHIs also targeted a range of mental health conditions, with the most common being mood-related (incl. [postnatal] depression, mixed depression/anxiety, and bipolar) (*n* = 9; 31.0%), transdiagnostic/mixed conditions (*n* = 8; 27.6%), or psychosis/schizophrenia (*n* = 6; 20.7%) (see Fig. 3, bottom).

**Fig. 4:**
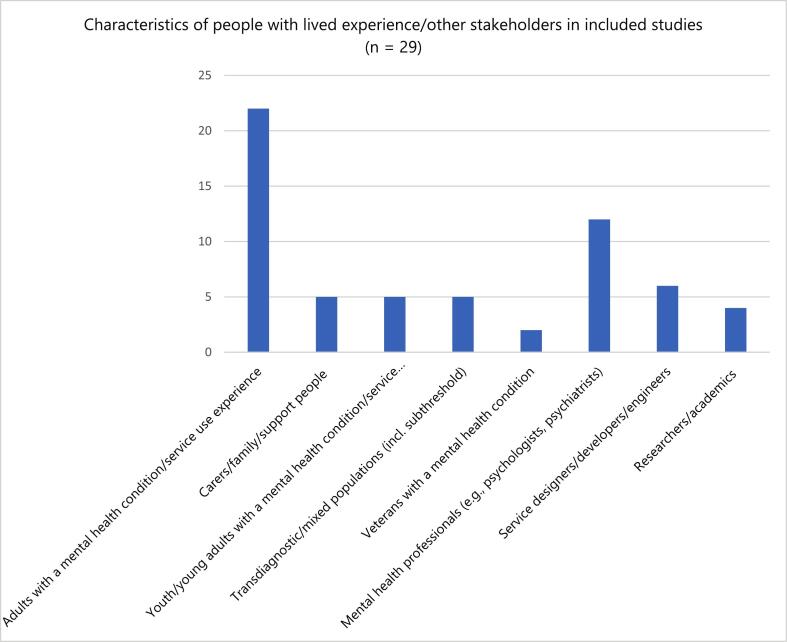
Characteristics of people with lived experience and other stakeholders in included studies (n = 29).

Across the included studies, engagement primarily involved PwLE of a mental health condition (and/or related service use) and their carers, most commonly adults with or supporting someone with depression, anxiety, psychosis, bipolar disorder, ADHD, eating disorders, PTSD, and postpartum depression (see Fig. 4). Engagement was largely adult-focused (typically ages 18–70) (*n* = 22; 75.9%), with some inclusion of young people and young adults (approximately 16–30 years), and older adults as a distinct group. Sample sizes varied widely by study and stage, ranging from small qualitative and advisory groups (n ≈ 2–20) to larger usability, survey, or trial-linked samples (up to *n* > 900), with engagement generally aligned to the intended target users of each DMHI. In addition to PwLE, most studies (*n* = 21; 72.4%) reported engaging other stakeholder groups with relevant professional experience. On over half of occasions (*n* = 12; 41.4%), these groups were mental health professionals with experience working with the DMHI's target population or condition, and/or significant content knowledge (e.g., expertise in CBT). For full details see Supplementary Table 4.

**Question 4:** Reported change/s made to DMHIs developed with engagement of PwLE.

In almost all studies (*n* = 26; 89.7%), authors reported on changes or decisions made to the design, content, format, or delivery of the DMHI due to engaging PwLE and other stakeholders. In most cases, it was not possible to attribute changes or decisions specifically to the engagement of PwLE versus other stakeholders as changes and decisions tended to implicate multiple aspects of the DMHI (e.g., design and content) with the general aim of making the DMHIs more user-centred, inclusive, and relevant.

As detailed in Supplementary Table 5, changes to the DMHI design included: i) changes to the user interface/look, navigation and layout (Abraham et al., 2018; Callan et al., 2021; Guala et al., 2023; Hughes-Barton et al., 2023; Lederman et al., 2019; McClelland and Fitzgerald, 2018; Sin et al., 2019); ii) use of colour, imagery and sounds (Hughes-Barton et al., 2023; McClelland and Fitzgerald, 2018; Sin et al., 2019; Geerling et al., 2022; Wiberg et al., 2022); and iii) branding input such as names and logos (Hughes-Barton et al., 2023; Sin et al., 2019; Ben-Zeev et al., 2013; Midgley et al., 2021). Changes to the DMHI content included: i) revised language, wording use and tone, such as simplifying, removing jargon and more inclusive language (Geerling et al., 2022; Behr et al., 2024; Danaher et al., 2012; Geraghty et al., 2016; Lehavot et al., 2021; Patterson et al., 2022); ii) adaptations to improve the relevance to specific populations or cultural contexts (Abraham et al., 2018; Hughes-Barton et al., 2023; Lederman et al., 2019; Midgley et al., 2021; Behr et al., 2024; Lal et al., 2020; Ospina-Pinillos et al., 2019); iii) additional resources, information or examples (Hughes-Barton et al., 2023; Lehavot et al., 2021; Lal et al., 2020; Hidalgo-Mazzei et al., 2016; Reupert et al., 2020; Whiteside et al., 2019); and iv) inclusion of safety/crisis planning protocols or other personal support features, such as wellbeing tracking (Hughes-Barton et al., 2023; McClelland and Fitzgerald, 2018; Patterson et al., 2022). Regarding the format and functionality of DMHIs, changes were around: i) inclusion of or refinements to multimedia/audiovisual elements (Behr et al., 2024; Ospina-Pinillos et al., 2019; Flobak et al., 2021; Honary et al., 2018); ii) greater personalisation or tailoring of in-built features (McClelland and Fitzgerald, 2018; Geerling et al., 2022; Behr et al., 2024; Patterson et al., 2022); and ii) development of interactive features, such as chat forums, mood and goal tracking (McClelland and Fitzgerald, 2018; Honary et al., 2018; MacKinnon et al., 2022). In terms of the DMHI delivery, engaging PwLE (and other stakeholders) was reportedly accompanied by: i) developing or optimising the integration of coaching and support (Danaher et al., 2012; Patterson et al., 2022); ii) enhancements to accessibility and usability, such as clearer instructions or audiovisual elements (Hughes-Barton et al., 2023; Wiberg et al., 2022; Behr et al., 2024); and iii) the development of guiding principles and program logic (Geraghty et al., 2016). See Supplementary Table 5 for full details.

**Question 5:** Reported benefits or positive impacts of engaging PwLE.

In around two-thirds of studies (*n* = 18; 62.1%), the authors reported on a range of (their perceived) benefits or positive impacts following engagement with PwLE in the design and development of DMHIs (see Supplementary Table 5). These were based on the reported views and experiences of the authors, as opposed to those of PwLE/other stakeholders or measured impacts.

Authors related the engagement process to content and features (e.g., games, multimedia) that were more relevant, relatable and engaging (Wiberg et al., 2022; Midgley et al., 2021; Geraghty et al., 2016; Patterson et al., 2022; Torok et al., 2022) including for specific groups, such as veterans and youth (Abraham et al., 2018; Guala et al., 2023; Lehavot et al., 2021; Flobak et al., 2021). According to authors, engagement was helpful in identifying and aligning DMHIs with people's real-world lived experiences, preferences and needs, and was seen to improve DMHI credibility, trust, acceptability, appropriateness, and (potential) usefulness among end-users (incl. Consumers and referrers) (Hughes-Barton et al., 2023; Wiberg et al., 2022; Danaher et al., 2012; Lehavot et al., 2021; Honary et al., 2018; Torok et al., 2022). Some authors also argued that engagement led to the development of content and features that were more representative, culturally appropriate, and inclusive of diverse user groups (Ospina-Pinillos et al., 2019; Flobak et al., 2021). Other perceived benefits of engagement that were noted included mutual learning between professional and lived experience groups (Flobak et al., 2021) and practical insights around usability issues, barriers to use, and solutions for addressing these (Hughes-Barton et al., 2023; Ben-Zeev et al., 2013; Patterson et al., 2022; Lal et al., 2020). See Supplementary Table 5 for full details.

**Question 6:** Reported challenges, costs or negative impacts of engaging PwLE.

In slightly less than half of studies (*n* = 14; 48.3%), the authors reported on several (of their perceived) challenges, costs or negative impacts of engaging PwLE in the design and development of DMHIs (see Supplementary Table 5). Again, as in Question 5, these were based on the reported views and experiences of the authors, as opposed to those of PwLE/other stakeholders or measured impacts.

A number of authors reported that their engagement of PwLE may have been limited by small or non-diverse groups (e.g., predominantly white, heterosexual, women), whose perspectives may not have been representative of or applicable to the broader population (Callan et al., 2021; Sin et al., 2019; Geerling et al., 2022; Behr et al., 2024; Danaher et al., 2012; Patterson et al., 2022; Torok et al., 2022; Batchelor et al., 2022). In addition, authors reported challenges in reconciling divergent or conflicting viewpoints, whether these arose between different stakeholder groups (e.g., professional versus lived experience) (Hughes-Barton et al., 2023; Behr et al., 2024; Flobak et al., 2021), individuals' personal views versus the group overall (Geerling et al., 2022), or between different individuals (Lal et al., 2020).

Other challenges reported by authors were more practical and related to difficulties scheduling in and retaining PwLE (Abraham et al., 2018; Patterson et al., 2022), issues posed by technology (sharing of personal stories online, unreliable internet connection) (Honary et al., 2018), as well as the time, cost, and resource demands of engagement, which was said to require significant skills, expertise, patience, and flexibility from all involved parties (incl. The researchers) (McClelland and Fitzgerald, 2018).

**Question 7:** Reported (measured) outcomes of DMHIs developed with engagement of PwLE.

No studies in this review compared the outcomes of DMHIs developed with engagement of PwLE to the outcomes of DMHIs developed without PwLE. The results below describe the use of DHMIs, the attitudes towards using DHMIs, and/or the efficacy or effectiveness of the DHMIs on mental health symptoms among the studies that reported these outcomes (*n* = 22; 75.9%, see Supplementary Table 5). There was heterogeneity in how outcomes had been evaluated and measured, with both quantitative and qualitative findings reported. In addition, there were no studies directly comparing the outcomes of DMHIs developed with versus without PwLE engagement. As distinct from Questions 5 and 6, the outcomes described here were measured impacts, as opposed to the reported views and experiences of the authors.

In terms of use, participants reported high usability and ease of use (Callan et al., 2021; Wiberg et al., 2022; Ben-Zeev et al., 2013; Behr et al., 2024; Patterson et al., 2022; Reupert et al., 2020), along with high rates of continued use and completion of modules (Geerling et al., 2022; Wiberg et al., 2022; Hidalgo-Mazzei et al., 2016; MacKinnon et al., 2022; Milgrom et al., 2016). There was however some noted variability in uptake, with some evidence of it being high (MacKinnon et al., 2022) or low despite positive experiences reported by participants who used the DMHI (Terp et al., 2018).

The DMHIs were reportedly associated with moderate to high ratings for acceptability (Hughes-Barton et al., 2023; Geerling et al., 2022; Wiberg et al., 2022; Danaher et al., 2012; Patterson et al., 2022; Reupert et al., 2020; Flobak et al., 2021), satisfaction (Callan et al., 2021; Hughes-Barton et al., 2023; Wiberg et al., 2022; Lehavot et al., 2021; Patterson et al., 2022; Hidalgo-Mazzei et al., 2016; MacKinnon et al., 2022; Milgrom et al., 2016), perceived feasibility (Callan et al., 2021; Bucci et al., 2019), and usefulness/helpfulness (Callan et al., 2021; Guala et al., 2023; Geerling et al., 2022; Ben-Zeev et al., 2013; Hidalgo-Mazzei et al., 2016; MacKinnon et al., 2022; Batchelor et al., 2022; Milgrom et al., 2016). The DMHIs were also described in positive terms by participants, who saw them as credible, supportive, relatable, and relevant (Lederman et al., 2019; Behr et al., 2024; Reupert et al., 2020; Flobak et al., 2021; Batchelor et al., 2022), while allowing them to feel safe, respected and cared for (Reupert et al., 2020; Bucci et al., 2019), and confident to manage their mental health (Terp et al., 2018). Some reported negative attitudes towards using the DMHI included increased mental health-related anxiety, namely fear, worries and uncertainty (Terp et al., 2018).

The efficacy or impacts of using the DMHIs on mental health outcomes were less commonly reported. Where reported, intervention use was associated with improvements in a variety of symptom domains, and across both controlled (RCT) and uncontrolled (single-arm trial, qualitative) study designs. Improvements were reported for: depression and postnatal depression (Callan et al., 2021; Wiberg et al., 2022; Reupert et al., 2020; MacKinnon et al., 2022; Milgrom et al., 2016), anxiety (Midgley et al., 2021), suicidal ideation (Whiteside et al., 2019; Torok et al., 2022), stress (Reupert et al., 2020), eating disorders (Wiberg et al., 2022), and emotion regulation (MacKinnon et al., 2022).

Of these studies, some found limited or mixed evidence for intervention use and symptom improvements. For example, in their uncontrolled trial, Midgley et al. (Midgley et al., 2021) found no improvements for anxiety but did for depression and emotion regulation. Meanwhile, in their RCT, MacKinnon et al. (MacKinnon et al., 2022) found no greater improvements for depression in the intervention group relative to control, but did for secondary outcomes, anxiety and sleep. In other RCTs, intervention-related improvements in PTSD symptoms (Lehavot et al., 2021) and secondary mental health outcomes (Torok et al., 2022) were no greater than those for the control group.

Some adverse effects were also noted, with Wilberg et al. (Wiberg et al., 2022) reporting increases in stress and anxiety among intervention users in their uncontrolled trial.

## 4 Discussion

This scoping review of 29 studies described and synthesised the current available evidence and gaps with regards to the engagement of PwLE in the design and development of DMHIs. Included studies were all conducted in high-income countries and most reported consultation-level engagement. Study authors frequently reported on the benefits of PwLE engagement, as well as the changes to the content, design and delivery of the DMHIs made following engagement. However, the specific influence of engaging PwLE on DMHI outcomes remains unclear, especially in terms of efficacy and effectiveness at improving mental health outcomes.

### 4.1 Publication patterns of studies

All included studies were published between 2012 and 2024, with the highest numbers in the years 2019 and 2022. Of note, these high publication years coincide with pre- and post-COVID-related lockdowns and other physical distancing measures (in 2020 and 2021), which may have disrupted engagement and broader research processes, while also demonstrating the feasibility and effectiveness of DMHIs to upscale access to mental healthcare (Castro Sweet and Altman, 2022; Titov et al., 2020). Included studies were all conducted in high income countries, most notably Australia, the US, UK, and Canada. This mirrors prior reviews (Bevan Jones et al., 2020; Orlowski et al., 2015; Veldmeijer et al., 2023; Sheikhan et al., 2023; Hawke et al., 2023) and is not unexpected given strong top-down influences; namely, that major funding bodies in these countries now require and give substantial weighting to engaging PwLE in grant applications (NHMRC, 2016; National Institute for Health and Care Research, 2024; Patient-Centered Outcomes Research Institute, 2024). This concentration of lived experience engagement in research in high income countries justifies recent attention on developing DMHIs for lower resource settings (Faria et al., 2023). In these settings, access to mental health services, digital infrastructure, help seeking norms, and research funding differ substantially (Faria et al., 2023) and so developing DMHIs may require alternative, contextually-grounded approaches of PwLE engagement.

### 4.2 Levels and types of PwLE engagement reported across studies

Almost all studies reported “consultation”, which typically involved consumers with relevant lived experience of a mental health condition and/or service use, their family members/carers, as well as mental health professionals, researchers, and designers. Around half of studies reported “involvement” and “collaboration”, with a notable absence of studies employing lived experience-led approaches. As part of consultation, engagement activities more commonly included focus groups, interviews, and user testing and less commonly, advisory groups and expert panels. The predominantly consultative nature of engaging PwLE is consistent with prior reviews of engagement in digital mental health research (Bevan Jones et al., 2020; Orlowski et al., 2015; Veldmeijer et al., 2023). It is unknown though whether the predominance of consultation in this review and others stems from reporting that is unclear or lacking in detail (Jones et al., 2021). Despite this, one noted limitation of consultation is that it aligns engagement with more commercial and transactional approaches to design that focus on acceptability and usability testing (Orlowski et al., 2015; Clemensen et al., 2007), such as user-centred design (Bernaerts et al., 2024). While there is expert consensus among mental health and digital health professionals on the merits of user-centred design for developing DMHIs (Seiferth et al., 2023), it may not satisfy the core principles of participatory research and codesign, such as relationship building, mutual learning, power sharing, and shared decision-making control (Bernaerts et al., 2024; Hughes and Duffy, 2018; Kelly, 2019). Exemplifying these principles, is research in which PwLE are engaged early and often (or throughout) rather than at discrete time-points and hold genuine decision-making authority.

This said, almost half of the included studies also drew on different levels of engagement (e.g., “consultation” and “involvement”) at different points in their multistage development process. This suggests that engagement may be better thought of as a dynamic spectrum as rather than as a rigid hierarchy (Hughes and Duffy, 2018).This suggestion is further supported by the five included studies reporting engagement activities that appeared to sit between two adjacent levels rather than fitting neatly into one (Guala et al., 2023; Wiberg et al., 2022; Patterson et al., 2022; MacKinnon et al., 2022; Terp et al., 2018). In this way, there is no uniformly best level of engagement but rather a level – for example, consultation – may be more or less suited to a particular stage of development (or phases of the research more broadly) and complement other levels occurring at different stages. This accords with systematic findings from Vandekerckhove et al. (Vandekerckhove et al., 2020) showing different engagement methods occurring at different stages of digital intervention design and development, and to serve different purposes. For example, field work and interviews might be more conducive to understanding context and uncovering latent needs in the initial exploration and pre-design stage, where more “active” co-creation methods like workshops and storytelling might be more conducive to generating ideas and sharing diverse perspectives at the ideation and concept generation stage (Vandekerckhov et al., 2020). Decisions around the best suited level and/or type of engagement may be similar to other researcher decisions around the suitability of different research methodologies to address the research aims in a timely, rigorous, and ethical way (De Beurs et al., 2017). Indeed, these decisions around PwLE engagement (and any implications for the project) may be guided by the advice of PwLE, and practical suggestions for operationalising engagement under different circumstances (Jones et al., 2021; De Beurs et al., 2017).

### 4.3 Impacts of PwLE engagement: reported benefits and challenges

Most studies drew on the subjective views and opinions of the study authors on their perceived benefits and challenges of consumer engagement, which broadly corroborated those reported previously (Bevan Jones et al., 2020; Orlowski et al., 2015; Veldmeijer et al., 2023; Brotherdale et al., 2024). Authors commonly credited engagement with improvements in the DMHI's relevance, credibility, acceptability, and inclusivity, along with difficulties in limited participant diversity and/or representativeness, reconciling differing views, sustaining engagement, and high resource demands). The absence of pre-post- evaluations of the DMHI, prior to and following engagement mean that the author reported improvements cannot be directly attributed to engagement and thus do not demonstrate causality. In addition, the observed lack of formal evaluation measures in the included studies to report on the impacts of engaging PwLE in research has been a noted limitation elsewhere in the literature (Veldmeijer et al., 2023; Sheikhan et al., 2023; Hawke et al., 2023; Peters et al., 2024). To advance knowledge and shared learnings around the impacts of lived experience engagement, Peters et al. developed the Codesign Evaluation Framework for formally evaluating engagement processes and outcomes across multiple domains prospectively, concurrently, and/or retrospectively. As such, this framework has scope to evaluate, inform, and make ongoing refinements to planning and carrying out PwLE engagement. Meanwhile, using other standardised measures, such as the Patient Engagement In Research Scale (Hamilton et al., 2018; Hamilton et al., 2021), will generate important knowledge on the quality and impacts of engagement from a lived experience perspective, which was lacking in the included studies.

### 4.4 Impacts of PwLE engagement: reported changes to the DMHIs

Almost all included studies reported that PwLE engagement led to changes across multiple aspects of the DMHI design and delivery, such as changes to the user interface, navigation, and visual design elements, functionality, and adapted or additional content. Although reported changes were aimed at making DMHIs more user-centred, accessible and relevant, it was not always clear who was involved in deciding on which changes to make or not. Here, guidelines for more transparent and detailed reporting on lived experience engagement in research could help disentangle the specific contributions of PwLE versus other stakeholders, or the research team (Bernaerts et al., 2024; Smits et al., 2020; Staniszewska et al., 2017).

### 4.5 Reported outcomes of DMHIs developed with engagement of PwLE

The limited reporting of efficacy and effectiveness outcomes for DMHIs – compared use and attitudes towards using – appeared to be because evaluations had not been completed and/or published at the time of review searches. Alternatively, it could be that the research did not progress past the DMHI development stage, a commonly cited shortcoming in the digital health research literature (Orlowski et al., 2015; Bird et al., 2021). While almost uniformly positive findings were reported for the use and attitudes towards using DMHIs, especially on qualitative and mixed-methods measures, more mixed findings were reported for DMHIs' efficacy and effectiveness. Similar to a review of codesigned in-person interventions (Barker et al., 2024), some studies reported null (Midgley et al., 2021; Lehavot et al., 2021; MacKinnon et al., 2022; Terp et al., 2018) or adverse findings (Wiberg et al., 2022) on mental health outcomes. Given the heterogenous nature of the evidence in terms of intervention types and study designs, it is not possible to attribute DMHI outcomes to PwLE engagement specifically. Moreover, the lack of head-to-head comparisons between DMHIs with and without PwLE engagement precludes any firm conclusions regarding improvements in outcomes. Future research that seeks to determine the specific effects of engaging PwLE will help address these current knowledge gaps. To this end, future research that draws on the complementary strengths of controlled quantitative (e.g., RCTs, quasi-experimental) and semi-structured qualitative study designs will help ensure comprehensive insights into PwLE engagement (Sheikhan et al., 2023; Bernaerts et al., 2024; Peters et al., 2024).

### 4.6 Limitations

In deciding to include studies and extract relevant study data, we relied solely on information reported in the published or associated articles. It is therefore likely that other studies engaging PwLE in the design and development of DMHIs were either not included or not described fully in this review. Indeed, most articles were excluded at the full text review stage (*n* = 399; 83.0%) because there was no clear indication that the researchers had engaged PwLE beyond the role of participants providing data. However, the potential value of formally including PwLE in intervention development, and therefore associated reporting, appears to be a relatively new phenomenon. More consistent use of terminology, along with standardising methods for carrying out and reporting on engagement would help ameliorate these issues (Sheikhan et al., 2023; Bernaerts et al., 2024; Peters et al., 2024). Of note, as a scoping review, this review did not include any assessment of the quality of included studies (Peters et al., 2020; Munn et al., 2018). Any empirically derived recommendations or guidelines for future research and practice would need to consider the quality of the available evidence. Also warranting consideration are potential limits to the generalisability of the current findings, given that PwLE engagement in the included studies saw an over-representation of certain groups (e.g., white women) and countries (high income, Western, developed nations). Next, due to time- and resource-constraints, full-text screening, data extraction, and classification of engagement levels were completed by a single reviewer. This may introduce some unintentional bias, which we tried to mitigate in several ways, namely: piloting both the search criteria and data extraction forms; involving more than one reviewer at the title-abstract screening stage; having a second reviewer independently check a random sample of extracted study data; and choosing a framework with explicit descriptions of engagement levels/types along with illustrative examples. Future systematic reviews may choose to minimise potential bias by including more than one independent reviewer throughout. Finally, given the fast pace of growth in the field, it is possible that relevant studies have been published in the period since the current searches were run (April 2024). This said, the current searches were comprehensive, covering over 23 years, and the resultant findings can be used to inform directions for a future, more exhaustive systematic review.

### 4.7 Practical implications

As a scoping review, the current findings are intended mainly to map out the breadth of available evidence, identify gaps, and guide future research priorities. That said, the current findings may inform researchers' planning, resourcing, and reporting of PwLE engagement in the design and development of DMHIs. In terms of planning, researchers are encouraged to plan PwLE engagement as a stage-specific and flexible process, selecting and justifying different engagement levels and methods according to the aims and phase of DMHI development, rather than assuming a single “best” approach. When considering resourcing, researchers need to anticipate and resource the (greater) time and effort demands of PwLE engagement, including sustaining involvement and supporting diversity, which were commonly reported as challenges. Finally, in terms of reporting researchers need to make efforts to improve transparency and consistency in reporting by clearly describing who was engaged, at what stages, using which methods, and how engagement informed decisions, drawing on existing engagement and evaluation frameworks.

## 5 Conclusion

This scoping review shows increasing engagement of PwLE in the design and development of DMHIs. While engagement was commonly credited with improvements in relevance and acceptability, most studies relied on consultative approaches to engaging PwLE. Transparent reporting and robust evaluations of engagement were lacking, precluding any conclusions about specific links between engagement and DMHI outcomes. By addressing the limitations highlighted by this review, researchers and funding bodies will be better informed and positioned to harness the unique contributions of lived experience expertise and more effectively deliver on the promise of DMHIs to support accessible, evidence-based mental healthcare that meets the needs and preferences of the people intended to benefit.

## CRediT authorship contribution statement

**Alana Fisher:** Conceptualisation; Data curation; Investigation (title/abstract screening; full-text screening; data synthesis); Methodology; Project Administration; Supervision; Visualisation; Writing – original draft; Writing – review & editing.

**Noni Jervis:** Investigation (title/abstract screening; data extraction); Writing – review & editing.

**Madelyne Bisby:** Investigation (title/abstract screening); Methodology; Writing – review & editing.

**Milena Gandy:** Investigation (title/abstract screening); Methodology; Writing – review & editing.

**Andreea I Heriseanu:** Investigation (title/abstract screening); Methodology; Writing – review & editing.

**Taylor Hathway:** Investigation (title/abstract screening; data extraction); Writing – review & editing.

**Atria Rezwan:** Conceptualisation; Writing – review & editing.

**Nickolai Titov:** Methodology; Supervision; Writing – review & editing.

**Blake Dear:** Conceptualisation; Methodology; Supervision; Writing – review & editing.

## Consent to participate

Not applicable as a scoping review of published studies.

## Consent for publication

Not applicable as a scoping review of published studies.

## Ethical considerations

Not applicable as a scoping review of published studies.

## Funding

This research was partly supported by a 10.13039/501100001230Macquarie University Research Fellowship, held by the first author (AF).

## Declaration of competing interest

The authors declare the following financial interests/personal relationships which may be considered as potential competing interests: Alana Fisher reports financial support was provided by Macquarie University. Alana Fisher reports financial support was provided by Macquarie University. Alana Fisher reports a relationship with Macquarie University that includes: employment. If there are other authors, they declare that they have no known competing financial interests or personal relationships that could have appeared to influence the work reported in this paper.

## Data Availability

Not applicable as a scoping review of published studies.

## Appendix A Supplementary data

Supplementary tables Image 1
