# Supplementary material for: Engagement of people with lived experience in the design and development of digital mental health interventions: A scoping review of engagement characteristics and impacts
Source: Internet Interv. 2026 Jan 29;43:100914. doi: 10.1016/j.invent.2026.100914 (PMC12877841; doi:10.1016/j.invent.2026.100914)
Supplement: Supplementary file 1 — Supplementary tables [file mmc1.docx]

**Supplementary Table 1.** Search strategy example (MEDLINE)

| **Concept** | **Line** | **Search terms** |
| --- | --- | --- |
| **Concept 1 - Lived experience in research** | **1** | Patient Participation/ or Community Participation/ or Advisory Committees/ |
|  | **2** | (patient* or client* or public or “service user*” or youth or consumer* or citizen*) adj2 (participat* or engag* or invol*).mp |
|  | **3** | “liv* expertise” or “lived experience” or “peer* researcher*” or “co‐researcher*” or “expert* by experience*” or “patient* partner*” or “patient* advisor*” or “co‐produc*” or “co-develop*” or “co‐design” or consult* or collaborat* or participatory.mp |
|  | **4** | design* or develop* |
|  | **5** | **1 or 2 or 3 or 4** |
| **Concept 2 - Mental health/illness** | **6** | Mental Health/ or Mental Disorders/ |
|  | **7** | (mental* or psychiatr* or psycholog*) adj2 (health* or ill* or hygiene or disorder* or distress* or condition*).mp |
|  | **8** | **6 or 7** |
| **Concept 3 - Digital interventions** | **9** | Internet-Based Intervention/ or Mobile Applications/ or Therapy, Computer-Assisted/ |
|  | **10** | (online or digital or mobile or smartphone or web-based or or web-delivered or internet* or computer* or cyber or electronic or virtual) adj4 (intervention* or treatment* or therap* or tool* or program*) |
|  | **11** | (mobile AND program*).mp |
|  | **12** | (mhealth or ehealth or mtherap* or etherap* or telehealth or telemedicine or teletherap* or “mobile app*”).mp |
|  | **13** | **9 or 10 or 11 or 12** |
|  | **14** | **5 and 8 and 13** |
| **Limits** | **15** | **Limit 14 to published 2000 onwards** |

**Supplementary Table 2:** Eligibility criteria using PICOS framework

|  | **Inclusion criteria** | **Exclusion criteria** |
| --- | --- | --- |
| **Population** | 1. People (aged 16 years and older) with a personal lived/living experience of:   - mental health distress (i.e., consumers)  **AND/OR**  - supporting someone with mental health distress (i.e., caregivers/family members/kin).   1. Engaged in the design/development of an intervention (see below).   **NB: Engagement** denotes actively involving people to a greater extent than as participants in research. | 1. People whose lived/living experience relates mainly to a non-mental health condition (incl. neurological or neurodevelopmental, alcohol/other drug use condition). |
| **Interventions** | 1. An intervention that is:  - Digital, i.e., a tool or program that is at least partially delivered online using an internet-connected device (e.g., laptop/desktop, smartphone, tablet).   **AND**   - Designed to treat, improve, manage or prevent mental health symptoms or conditions, psychological distress, emotional well-being, (symptoms may or may not meet diagnostic/clinical thresholds). - **AND** - Includes a combination of psychoeducation and psychosocial/psychological support, skills or guidance. - **AND** - Designed for people aged 16 years and older. | 1. No intervention 2. Discussion of a category of interventions (e.g., attitudes towards/use of therapist-guided iCBT for depression) 3. Interventions not specific to mental health, but instead:  - Pharmacology - physical health or “lifestyle behaviours” (e.g., activity/exercise/nutrition) - other conditions/comorbidities (e.g., insomnia, alcohol/other drug use, neurological or neurodevelopmental, or chronic health conditions) - symptom monitoring/tracking - assessment/detection/diagnosis or screening - stigma or attitudes towards mental health/help-seeking - mental health literacy/training - factors associated with mental health difficulties (e.g., perfectionism, gratitude, self-compassion, self-efficacy, self-esteem, loneliness, alexithymia, resilience, burnout). |
| **Comparison/Control group** | 1. Studies with and without a control group | N/A |
| **Outcomes** | 1. Intervention/treatment development outcomes (i.e., decisions around or changes to the intervention design, content, format, delivery) 2. Intervention/treatment outcomes related to:  - use (i.e., uptake/engagement, adherence/completion, feasibility of use) the intervention   **AND/OR**   - end-user attitudes towards using (i.e., acceptability, satisfaction).   **AND/OR**   - regarding efficacy or effectiveness (i.e., improvements in mental health or psychological symptoms).  1. Other reported research benefits, challenges or costs that are linked to engaging people with lived experience. | N/A |
| **Settings** | 1. Studies conducted in a research setting (e.g., University, research institute or clinic). | 1. Studies conducted in a routine care setting (i.e., with a focus on service development, evaluation and/or quality improvement). |
| **Types of articles to include** | Primary research studies in English published 2000 – 2024, including:  **✓** Randomised controlled trials (and other experimental studies) **✓** Other trials (e.g. uncontrolled trials; open/single-arm trials; feasibility trials; pre-test/post-test designs)  **✓** Observational studies (e.g., cross-sectional surveys, cohort designs) **✓** Quantitative and qualitative studies (including mixed-design and qualitative only) | ❌ Non-peer-reviewed, or unpublished studies or articles  ❌ Literature reviews of any type (narrative, systematic, scoping, meta-analysis).  ❌ Research dissertations ❌ Protocol papers ❌ Conference abstracts or papers ❌ Case report or series ❌ Editorials or commentaries ❌ Animal studies |

**Supplementary Table 3.** Article characteristics for data extraction

- **Study authors**
- **Year of publication**
- **Country of origin**
- **Study aims/purpose**
- **Study type/design** (e.g., RCT, open trial, cohort study, qualitative study).
- **DMHI type** (e.g., mobile app, self-guided, therapist-guided treatment)
- **DMHI target population/condition** (e.g., depression, anxiety, youth, older adults)
- **Characteristics of the people with lived experience (e.g.,** sociodemographic, number and type/s of consumer/carer/family member, mental health experience/s).
- **Engagement of other stakeholder or community members** (e.g., health professionals/clinicians).
- **Level/s of engagement**
- **Type/s of engagement activities** (e.g., surveys, interviews, focus groups, workshops, reference groups/advisory committees, co-investigators and research partners, principal investigators).
- **Research phases with engagement** (e.g., design/development, testing of the intervention)
- **Reported positive impacts or benefits of engagement**
- **Reported negative impacts, challenges or costs of engagement**
- **Reported impacts of engagement on intervention development** (e.g., changes or decisions made to the design, content, format, or delivery of the intervention).
- **Reported impacts of engagement on intervention outcomes** **(if tested/measured).**
  1. **use:** uptake/engagement, adherence/completion, feasibility of use
  2. **attitudes towards using:** acceptability, satisfaction
  3. **efficacy or effectiveness of the DMHI:** improvements in mental health or psychological symptoms.

**Supplementary Table 4.** Overview of study characteristics and description of activities engaging people with lived experience (PwLE)

| **Study details** | **Study aims/purpose** | **Study type/design** | **DMHI type** | **DMHI target population/condition** | **Characteristics of PwLE** | **Engagement of stakeholders or community members** | | **Level/s of engagement** | **Type/s of engagement activities** | **Intervention development phase/s with engagement of PwLE** |
| --- | --- | --- | --- | --- | --- | --- | --- | --- | --- | --- |
| Abraham *et al*. (2018), USA | - To use feedback from key stakeholders to adapt and develop a computer-based program to rural Veterans Affairs Community Based Outpatient Clinics - To determine the acceptability and feasibility of adapted program | Qualitative (iterative approach) | - Computer-delivered/internet-based program; - Adaptation of the CALM program for Veterans Affairs outpatient settings; - CBT framework (psychoeducation, cognitive restructuring, goals setting, exposure and response prevention); - Provider^a^ and patient^a^ complete together | Veterans with anxiety, PTSD and depression attending rural Veterans Affairs clinics | Adult veterans currently diagnosed with anxiety and depression. (Receiving recent mental health care from a Veterans Affairs Community Based Outpatient Clinic ) (n=11). | - Community Based Outpatient Clinics mental health providers (n=11); - “Expert CBT Veterans Affairs clinicians” (n=6) - Veterans Affairs “Central Office leaders” with “expertise in the implementation of evidence-based psychotherapies” within Veterans Affairs (n=5) | | Consult | - Focus groups over three stages (each group of key stakeholders participated) - Panel of experts decided on the recommendations that were feasible to action following analysis of focus group discussions - **Stage 1:** Review of existing CALM program: Stakeholders included: Veterans (n=11); Mental health providers (n=11); Veterans Affairs expert CBT clinicians (n=6); Veterans Affairs Central Office leaders (n=5) - **Stage 2:** Review of wireframe prototype: Stakeholders included Veterans (n=6), Community Based Outpatient Clinics mental health providers (n=10), expert clinicians (n=5) and Veteran Affairs leaders (n=5) - **Stage 3:**  Prototype pilot demonstration: Stakeholder groups Community Based Outpatient Clinics mental health providers, expert CBT clinicians and Veterans Affairs leaders - Veteran stakeholders (n=4) reviewed modified CALM modules and an overview of the modifications | Intervention development (adaption of existing program) |
| Batchelor *et al.* (2022), UK | - To explore the experiences of carers while using the COPe-support: including acceptability and impact of engaging with COPe-support - To explore carers ideas to inform improvements of COPe-support and its implementation | Qualitative (within a larger RCT reported separately) | - Internet-based platform: COPe-support (Carers for People with Psychosis e-support) - Multiple components: psychoeducation, guidance on well-being promotion information and exercises, links to external resources, moderated forums (one with a panel or experts and one with peers). - Included weekly email update for 4 months | Carers for people with Psychosis | Adult family members, relatives or close friends of someone diagnosed with psychosis (>20 years) (participating in the intervention arm of the RCT) (n=35) | N/A | | Consult (Interviews)  and Involve (Project Reference Group) | - Semi structured interviews with carers (n=35) following their use of the COPe-support as part of a RCT - Project Reference Group (included people with lived experiences of psychosis or caring for a loved one with psychosis) developed the interview guide | Intervention development and delivery (to inform future refinements and implementation) |
| Behr *et al.* (2024), Germany | To describe the process of developing TONI an internet-based intervention | Qualitative, (iterative approach) | - Internet-based program: TONI (Online modules ("my size fits me")) - Transtheoretical (CBT, psychodynamic and systemic) and transdiagnostic - For blended care settings | Adults with mental disorders (transdiagnostic) | Adults with a lived experience of mental illness (n=10) | Psychotherapists: Systemic (n=9) and Psychodynamic (n=20) | | Consult | - 2 x Focus groups with PwLE - Focus groups 1 (n=10). Focus group 1 informed development of intervention prototype - Focus group 2 collected feedback on prototype - Participants tested the interactive prototype and provided feedback on their user experience (System Usability Scale) - PWLE (n=2) proofread final texts. - Participants completed written feedback sheets | Intervention development |
| Ben-Zeev *et al.* (2013), USA | To describe development of a smartphone illness self-management system for people with schizophrenia | Mixed methods (Iterative approach) | - Smartphone self-management system: FOCUS - Designed to support self-management of illness - Includes applications with psychosocial intervention techniques for 1) Medication adherence, 2) Mood regulation, 3) Sleep, 4) Social functioning, and 5) Coping with persistent auditory hallucinations. - Users select what they focus on - Cognitive model of psychosis/stress-vulnerability model of schizophrenia | Adults with schizophrenia | - **Stage 1:** Adults with a diagnosis of schizophrenia or schizoaffective disorder (n=904) - **Stage 2:** "Consumers of psychosocial services" - **Stage 3:** Adults with a diagnosis of schizophrenia or schizoaffective disorders (n=12) | - **Stage 1:** Practitioners representing a range of specialisations and service models (n=8) - **Stage 2:** Practitioners (details not reported) | | Consult | - **Stage 1:** Survey on device use and interest in mHealth services (individuals with schizophrenia or schizoaffective disorder and practitioners) - Group discussion (practitioners only) - **Stage 2:** Practitioners and "consumers of psychosocial services" were “consulted throughout the development process” - **Stage 3:** 2 hr individual usability testing: participants could provide commentary during use of FOCUS - Stage 3 was conducted in two cycles and modifications were made to the intervention after each cycle. Cycle 1 (n=7), Cycle 2 (n=5) - Participants provided feedback on the FOCUS interface and engaged in the treatment modules - Participants completed a questionnaire and were asked to vote on the name of the system | Intervention development |
| Bucci *et al.* (2019), UK | To describe the development of a CBT informed app for early psychosis (Actissist) | Mixed methods (iterative approach). Beta-testing of the prototype version and proof-of-concept RCT. | - App for early psychosis: Actissist - CBT informed - Includes coping strategies, normalising, information, motivational interviewing techniques, psychoeducation, activity scheduling, mindfulness, relaxation exercises, recovery videos, resilience building, behavioural experiments, safety behaviour work, cognitive restructuring, interactive fact sheets | People registered with an early intervention for psychosis service who are within five years of experiencing a first episode of psychosis | - **Expert Reference Group:** "Service users" (People who have experienced a first episode of psychosis and who attend an early intervention for psychosis service) (n not reported) - **Beta-testing stage:** "End users" (n=10) | Expert Reference Group comprising of (in addition to service users) clinicians, software engineers, and clinical academics (n not reported) | | Consult (Interviews) and Involve (Expert Reference Group) | - App development: The Expert Reference Group met at the beginning and every three months of the project to discuss the app and potential refinements. - The clinical team rated the Expert Reference Group suggestions based on priority and feasibility - Validation study: Beta-testing with 10 “end-users” of prototype version | Intervention development and beta-testing |
| Callan *et al.* (2021), USA | To describe the development of the CBT MobileWork app.  Evaluate the feasibility, functionality, and usability of the app | Mixed methods (iterative approach). Prototype testing and open trial | - App: CBT Mobile Work - Promotes CBT skills practice | Adults with depression | Adults with a diagnosis of unipolar major depression receiving CBT (18-70 yrs) (n=8) | CBT therapists (n=6) | | Consult | - **Stage 1:** testing of the prototype with CBT therapists and patients. - Iterative design approach:  Session1: Patients viewed components of the app and completed an interview. Patients and therapists rated elements of the app. Session 2 and Session 3 participants (patients and therapists) trialled the app prototype and were asked to “think aloud”. - **Stage 2:** “real world testing”: patients used the app with therapist, followed by patient evaluations of app usability (during CBT treatment) | Intervention development and testing/revision |
| Danaher *et al*. (2012) USA and Australia | To describe the development and components of MomMood Booster, a web-based intervention for postpartum depression | Qualitative (iterative approach) | - Internet-based program: MomMoodBooster - Based on an adaption of group CBT treatment for postpartum depression. - Mothers also received Personal Coach calls. - Six sequential sessions scheduled across a 6-week period. - Intervention also included a partner support website and an administrative website (including a dashboard for Personal Coaches). | Mothers with postnatal depression | Adult mothers with postpartum depression (within 12 months post-partum)   - **Stage 1:** Focus groups (n=17). - **Stage 2:** Usability test (n=22). | N/A | Consult | | - Focus groups and usability testing session x 2: Melbourne (n=8); Iowa (n=9). - **Stage 1: Focus groups:** participants could comment on the proposed structure and content of the intervention, and mock-up of webpages. - **Stage 2: Usability tests:** Participants used components of the program and were asked to “think aloud”. - **Iterative process:** Feedback incorporated into program development. | Intervention development (adaption) |
| Flobak *et al.* (2021), Norway | To describe the process of development of an online intervention for adults with ADHD, My ADHD.  Evaluate My ADHD. | Qualitative (analysis of feedback from clinical trial) | - Internet-based program: My ADHD - 7 modules (psychoeducation, skill building exercise and coping techniques) for adults with ADHD. - Includes 2 video vignettes of coping techniques. | Adults with ADHD | - **Stage 1:** Adults with ADHD (n=12); - **Stage 2:** Adults with ADHD: Participants (n=109) and subgroup (n=7) completed interviews | **Stage 1:** Clinicians (n=2), Research assistants (n=2) | | Involve | - **Stage 1:** Prior to development: Meetings and workshops (n=3) - Co-production of video vignettes (n=12); - **Stage 2:** Clinical trial with feedback, followed by a subgroup of participants completing interviews | Intervention development |
| Geerling *et al.* (2022), Netherlands | - To explore views of people with bipolar disorder and health care professionals about online Positive Psychology Interventions for bipolar disorder - To develop and pilot-test a Positive Psychology Interventions based app for patients with bipolar disoder. | Qualitative (pilot test of the app; usability testing) | - App: Wellbeing Bipolar Disorder. - Based on Positive Psychology - 7 exercises in the 4 domains of Positive Psychology. | Adults with bipolar disorder | Adults with bipolar disorder I or II:   - **Stage 1**: (n=8); - **Stage 2:** (n=10) | Professionals (psychiatrists, psychologists, psychiatric nurses):   - **Stage 1**: (n=5); - **Stage 2:** (n=9). | | Consult | - **Stage 1:** 3x 2-hour focus group discussions: (opinions of Positive Psychology Interventions, requirements for the design of the app). - Prototype test. - **Stage 2:** App pilot test | Intervention development and testing |
| Geraghty *et al.* (2016), UK | To describe development of a web-based intervention, "Healthy Paths Through Stress" (Short name: Healthy Paths), designed to support primary care patients in reducing emotional distress. | Qualitative, (iterative approach) | - Internet-based program: Healthy Paths Through Stress - Unguided - Designed to support reduction of emotional distress in primary care setting. | Primary care patients with subthreshold depressive symptoms. | - **Stage 1:** Qualitative interviews: “Adults with distress” (n=20), - **Stage 2:** “Think aloud” qualitative interviews (n=13 from previous sample) | N/A | | Consult | - **Stage 1:** 1- 1.5-hour exploratory interviews; - **Stage 2:** Participants viewed an early prototype version while being asked to “think aloud”. | Intervention development |
| Guala *et al.* (2023), Denmark | - To explore patient and therapist experiences of a digital serious game designed for eating disorders, Maze Out. - To examine the acceptability of Maze Out. | Qualitative (pilot study) | - Digital serious game for tablet or smartphones: Maze Out - Participants complete 10 missions through the maze to find their way out. - Includes different themes: (challenges relate to food and exercise, feelings, relationships and communication). - Participants can also perform reflection exercises. | People with eating disorders | - **Stage 1:** Intervention development. Adults with eating disorders (n=4) - **Stage 2:** Evaluation of Maze Out: Adults (21-45 yrs) with an eating disorder: (n=20). | - **Stage 1:** Therapists  (n=3) and a commercial game company; - **Stage 2:** Therapist  (n=1). | | Consult and Involve/  Collaborate | - Six 4-hour face to face workshops - Regular mail correspondence between members of the “coproduction team” - Participation in a private chat forum after each iteration to discuss amendments - Joint decisions on the content were made by the team during workshops. | Intervention development and testing |
| Hidalgo-Mazzei *et al*. (2016), Spain | - To evaluate acceptability, safety and satisfaction of a smartphone application, SIMPLE - To explore predictors or enhancers of usage - To collate patient feedback to improve future versions | Single-arm feasibility trial | - Smartphone App: SIMPLe - Designed to monitor symptoms of bipolar disorder - Includes psychoeducation content encouraging self-management | Adults with bipolar disorder | - **Stage 1:** Patients. - **Stage 2:** Evaluation: Adult patients with bipolar disorders (type I, II or not elsewhere specified) | **Stage 1:** Development: International “field experts”, software engineers, and graphic designers. | | Consult | **Stage 1:** Development: Individual interviews, focus groups, online surveys and forums, alpha and beta test groups | Intervention development |
| Honary *et al.* (2018), UK | To develop a Web-based intervention to support relatives of people experiencing psychosis or bipolar disorder | Qualitative, (iterative approach) | - Internet-based program/toolkit: Based on the Relatives Education and Coping Toolkit Booklet (REACT) - Modular toolkit including information on management of symptoms, managing difficult behaviour, coping with their own stress, information about medication, and understanding mental health services | Relatives (family, friends and caregivers) of people with experiences of bipolar disorder or psychosis | - **Stage 1:** Relatives (family, friends and caregivers) of people with experiences of bipolar disorder or psychosis - **Stage 1:** 2-hour workshops 1 (n=13) and 2 (n=11); workshop 3 (n=2) - **Stage 2:** Testing in a controlled setting (n=3); Uncontrolled field testing (n=8) | N/A | | Consult and Involve | - **Stage 1:** Workshops and review of prototypes - **Stage 2:** Controlled setting testing: Participants used the intervention while being asked to “think aloud” - Uncontrolled field testing: Feedback via email | Intervention development (adaption of paper based REACT booklet) |
| Hughes-Barton *et al.* (2023), Australia | To explore consumers’ and health professionals’ views for the development of an online intervention, i can act now, for Australian adults. | Mixed methods (with prototype testing) | - Internet-based program: i can act now. - Based on ACT - Adapted from: ifarmwell website (co-designed with Australian farmers). - Transdiagnostic approach. - Modules include tips to improve ability to manage stress and prevent mental health issues, strategies for poor mental health, and guidance for people awaiting an appointment with a mental health professional. | General adult population (including people currently experiencing poor mental health). | - **Stage 1:** Part 1 Adult mental health service consumers (current or potential) and carers of people who had experienced poor mental health (n=14); - **Stage 2:** Consumers and carers (n=11); - **Stage 3:** Consumers and carers (n=8). The advisory panel also included consumers (n=3). | - **Stage 1:** Part 1: Mental Health professionals (n=2); GPs (n=3); Member of Advisory panel (n=1); Part 2: Mental Health professional (n=1); Member of Advisory panel (n=1). - **Stage 2:** Mental Health Professionals (n=2); Members of advisory panel (n=7). - **Stage 3:** Advisory panel members (n=2), Part 2: Mental health professional (n=1), GP (n=1), Member of advisory panel (n=1). | | Consult and Involve | - **Advisory panel:** Planning meetings prior to data collection to inform intervention scope and focus. - **Stage 1:** Semi-structured interviews on preferences/priorities. - **Stage 2:** Revision of website content. Participants could email feedback on branding options - **Stage 3:** Prototype testing: Part A: Participants viewed prototype and provided feedback. Part B: A separate group of participants viewed the updated prototype. Feedback provided via Zoom or an online survey. | Intervention development (Adaption of ifarmwell) |
| Lal *et al.* (2020), Canada | - To assess the initial acceptability of an internet-based intervention, Horyzons - To adapt the intervention for future pilot testing in Canada. | Mixed methods | - Internet-based program: Horyzons - Based on ACT - Adapted from Australian version | Canadian youth receiving services for first episode psychosis. | Adult (19-37 yrs) Consumers diagnosed with a psychotic disorder (n=11) | Clinicians (n=15) | | Consult (n=11) and Involve (n=2) | - Focus groups; feedback forms, surveys, reviewing modifications, interviews | Intervention development (development and adaption of Australian version) |
| Lederman *et al.* (2019), Australia | To report on the development of an online intervention for carers, Merdian, adapted from the Moderated Online Social Therapy (MOST) and the trial of this intervention. | Qualitative (included a feasibility trial of the intervention) | - Internet based program: Meridian. - Adaption of the Moderated Online Social Therapy (MOST) platform - Psychoeducation modules with accompanying "action" tasks, moderated online chat room | Carers (parents, carers, families, personal networks) of young people with mental illness | Adult carers (40-60 yrs) (n=20) of young people with mental health conditions | Clinicians, "client representatives", writers, technology designers, and graphic designers | | Consult | **Workshops (x2):** Carers viewed possible components of Meridian and discussed their thoughts | Intervention development (adaption) |
| Lehavot *et al.* (2021), USA | To evaluate the feasibility, acceptability, and efficacy of DESTRESS–WV on PTSD symptoms within an RCT | RCT | - Internet-based program: Delivery of Self Training and Education for Stressful Situations Women Veterans version (DESTRESS-WV) - CBT-based - Adaption of DESTRESS - 8-week program, including homework activities - Including coach calls once a week to monitor symptoms and safety, review progress, provide encouragement, review a particular skill or strategy when applicable, and address barriers to treatment | US Women Veterans with PTSD | US Women Veterans with PTSD (n=15 as per protocol article Lehavot, et al. 2017)^c^ | Expert clinicians (psychologists or social workers who had been providing PTSD care to women Veterans for at least one year; as per protocol article Lehavot, et al. 2017)^c^ | | Consult | Qualitative interviews (reported in protocol paper) Interview 1 (n=15), then Interview 2 with the same participants (n=12) to review the changes made and provide any additional feedback | Intervention development (adaption) |
| MacKinnon *et al.* (2022), Canada | To develop and pilot test an App-based psychoeducation and social-connection platform: Building Emotional Awareness and Mental Health (BEAM) | RCT | - App: Building Emotional Awareness and Mental Health Program Development (BEAM) Program - Transdiagnostic emotion-focused cognitive behavioural therapy alongside emotional focused parenting strategies. - 10 weeks - Weekly videos and activities, weekly telehealth group review sessions and private online forum - 5 core modules | Mothers of preschool children (aged 18-36 months old) with various mental health concerns (for trial recruited only depression) | **Parent advisory board:** Mothers with lived experience managing depression (n=10) | N/A | | Consult/ Involve | Parent Advisory Board asked for suggestions on mental health supports and sought ongoing input during the design of the program | Intervention development |
| McClelland & Fitzgerald (2018), UK | To ascertain the utility of a mobile app for mental health services users and clinicians | Qualitative | - App - Behaviour change app - Included: Information, mood tracking, a diary, a help button linked to personal support | Mental health service users and clinicians | Services users of an early intervention psychosis service (n=10) | Clinicians at the early intervention in psychosis service (n=8) | | Consult | Focus groups:   - **Stage 1:** Consultation with service users and clinicians - **Stage 2:** Participants viewed a mock up and provided feedback | Intervention development |
| Midgley *et al.* (2021), UK | To evaluate the feasibility, acceptability, and efficacy of D:OTS, an online psychodynamic treatment (adapted from Swedish version) for adolescents with depression | Uncontrolled single-arm trial | - Internet-based program: (D:OTS). - Based on psychodynamic therapy - Therapist supported - 8 Modules (videos and text on specific topics, worksheets, messages, chat session with a therapist) - Adapted from the Swedish version | Adolescents with depression | Young people (aged 16-21) with experience of using mental health services (n not reported) | N/A | | Consult | Participants viewed translated modules and worksheets; and provided feedback via group video call | Intervention development (adaption) |
| Milgrom *et al.* (2020), Australia | To evaluate the efficacy of the MumMoodBooster program for women with postnatal depression in a randomized controlled trial (RCT) | RCT | - Internet-based program: MumMoodBooster - CBT based - 6 sessions, - supported by telephone coaching - Adapted from Getting Ahead of Postnatal Depression Program | Women with postnatal depression | From linked development paper (Danaher et al, 2012, above): Mothers wLE of post-partum depressive episodes | See above: Danaher et al, (2012) | | See above: Danaher et al, (2012) | From linked development paper: Focus groups (Melbourne n=8 and Iowa n=9); usability testing (Melbourne n=14 and Iowa n=8) | Intervention development (adaption) |
| Ospina-Pinillos *et al.* (2019), Australia | - To design and culturally adapt the the Mental Health eClinic (MHeC) with Native Spanish speaking young people, supportive others and health professionals - To usability test the MHeC-S alpha prototype and collect data to develop a beta prototype - To translate and culturally adapt the MHeC-S self-report assessment and assess it’s face validity | Qualitative (included prototype testing) | - Internet-based platform/eClinic: Spanish Version of the Mental Health eClinic (MHeC-S), - Adaption of the MHeC - 5 main elements of the alpha prototype include: home page and triage system; online physical and mental health self-report assessment; dashboard of results and progress report; booking and videoconferencing system to enable video visits; personalized well-being plan | International students seeking mental health help (Native Spanish speaking, young people in Australia) | Native Spanish speaking young people (aged 16 to 30 years) living in Australia and attending headspace (headspace Australia's National Youth Mental Health Foundation provides early intervention mental health services and assistance) (n=17) | - “Supportive others” (family, friends, caregivers, coaches, teachers, or community members) (n=3) - Health professionals (n=12) | | Consult and  Involve | - **Stage 1:** Co-design workshops x 2 (10 x young people in total) - **Stage 4:** Participants viewed the alpha prototype while being asked to “think aloud” (young people n=7; “supportive others” n=3) | Intervention development (adaption) |
| Patterson *et al.* (2022), Canada | - To evaluate the usability and fidelity of a novel iCBT program for anxiety, Tranquillity - To adapt Tranquility to incorporate treatment for depression | Mixed methods | - Internet-based program: Tranquillity: - iCBT intervention. - Includes personalised support through video, phone, and in-app messaging with a web-based coach | Adults with co-occurring anxiety and depression | "Mental health-informed peers" ("first voice advocate" or have experience with mental health conditions in a near-peer role e.g. peer mentors) (n=6), adults | "CBT experts" (minimum of 5 years' experience delivering CBT for depression and anxiety in adults; licensed by a professional body) | | Consult/  Involve | 2 x Co-design focus groups, survey | Intervention development |
| Reupert *et al.* (2020), Australia | To evaluate the acceptability, safety and potential impact of the online intervention, mi.spot, for young people who have parents with a mental illness and/or substance use issue | Mixed methods (incl. uncontrolled single-arm trial) | - Internet-based program: mi-spot - Based on competence enhancement model - 6-week manualised program - Includes: Homework activities, peer forums, weekly mental health check-ins - Optional components: six 1-hour weekly facilitated sessions; one-to-one online counselling sessions - Moderated by facilitators (master's level psychology students) | Young adults (18-25) with a parent experiencing mental illness and/or substance use issue | Young adults (18-25) who have a lived experience of a parent with a mental illness and/or substance use issue (n unclear) | Information technology designers, researchers and clinicians | | Consult and  Involve | **Stage 1:** Delphi study (n=268 young people) to identify intervention features.  **Stage 2:** Participation in a reference group (young people, researchers, clinicians, IT professionals), which met over 36 months to develop the site (n not reported)  **Stage 3:** A series of feasibility trials (N=66): Semi structured interviews with participants and online facilitators | Intervention development |
| Sin *et al.* (2019), UK | To describe the design and development process of an e-Health intervention, COPe-support for carers of people with psychosis | Qualitative (iterative approach; prototyping, build/review of offline alpha and online beta versions of intervention) | - Platform and app: COPe-support (Carers of People with Psychosis e-support) - Modules include psychoeducation, caring strategies, social and service issues, wellbeing promotion strategies and further resources. - Included discussion forum and blog, “ask the experts” forum | Family carers for People affected by psychosis | - Individuals with a lived experience of psychosis and carers - Expert advisory group: Individuals with lived experience of psychosis (n=3); family members (n=3); clinician with personal experience of family caregiving (n=1). - Consultation with carers: (n=24) | Health care professionals, researchers, voluntary organisation workers and eLearning experts (n=14) | | Consult and Collaborate; expert advisory group and research team made decisions together) | - Prior preliminary study to inform current study: Focus groups with individuals with psychosis and carers. - Current study: - 4 x Coproduction workshops (expert advisory group) - 2 x consultation focus groups with carers (n=10; n=12), - Viewed intervention prototype and identified revisions (expert advisory group) | Intervention development |
| Terp *et al.* (2018), Denmark | To explore the use and experience of a smartphone app, MindFrame, by young adults recently diagnosed with schizophrenia | Qualitative | - Smartphone App: MindFrame - Self-management resources: including self-assessment, visualisation, early warning signs, triggers and alerts, action plan, medication overview, settings - Implemented as an add-on tool to regular OPUS care in 1 OPUS clinic in Denmark | Young adults diagnosed with schizophrenia | Young adults recently diagnosed with schizophrenia (for more details see Terp et al, (2016)^d^; Terp et al, (2017)^e^) | Health care providers, software designers | | Consult/  Involve (see Terp et al, (2016)^d^; Terp et al, (2017)^e^) | Interviews; Community of Practice (see Terp et al, (2016)^d^; Terp et al, (2017)^e^) | Intervention development |
| Torok *et al.* (2022), Australia | To compare the efficacy of the smartphone application, LifeBuoy, at reducing suicidal ideation severity to an attention matched control application | RCT | - App: LifeBuoy based on DBT: - Brief self-guided intervention - Based on DBT - Targeting reduction in severity of suicidal ideation - 7 modules: Including emotion regulation and distress tolerance skills | Young people experiencing suicidal ideation | Young people with a lived experience of suicide (n not reported) | N/A | | Consult^b^ | Focus groups and surveys | Intervention development |
| Whiteside *et al.* (2019), USA | To outline the development of NowMattersNow.org and evaluate its utility for individuals with suicidal ideation | Quantitative (incl. uncontrolled single-arm trial) | - Website: NowMattersNow.org - Online video based - Based on DBT. - Includes: information, examples and resources for managing suicidal thoughts and intense emotions | Patients with suicidal ideation and providers | **Development stage:** “Mental health consumers with suicidal experiences” (n not reported)  **Evaluation stage:** Survey responses from site visitors (n=3670includes mental health professionals (n=460)/other health care providers (n=308)) | **Evaluation stage:**  Mental health care providers (n=460) and other health care providers (n=308) | | Collaborate | Co-developed videos which involved talking about their experiences | Intervention development |
| Wiberg *et al.* (2022), Sweden | - To develop and evaluate usability of an online treatment for bulimia nervosa or binge eating disorder - To evaluate feasibility (including preliminary outcomes) within an open trial | Mixed methods (incl. single-arm, open trial) | - Internet-based program: NARA - Based on Enhanced CBT - “Guided self help” - Five modules: psychoeducation, case-formulation, daily self-monitoring of food intake, establishing eating plans and regular eating patterns, weekly weighing, alternative activities and problem solving | Patients with full or subthreshold bulimia nervosa or binge eating disorder | Adults with current or previous history of eating disorders:   - **Stage 1:** Development of ICBT-E: (n=14); - **Usability evaluation:** (n=24) | Clinicians (n=8) (clinicians working in the outpatient unit at Stockholm Centre for Eating Disorders) | | Involve/  Collaborate | - Focus groups x 4; - Think aloud observations x 16. - 8 stages of development:   1) development of prototype  2) evaluation  3) refine and develop  4) evaluation  5) refine, develop, and implement  6) ongoing feedback collected from users  7) refine and develop and  8) validate | Intervention development |

*Note* ACT = Acceptance and Commitment Therapy, ADHD = Attention Deficit Hyperactivity Disorder, CBT = Cognitive Behavioural Therapy, DBT = Dialectical Behavioural Therapy, DMHI = Digital Mental Health Intervention, iCBT = Internet-based Cognitive Behavioural Therapy, PTSD = Post Traumatic Distress Disorder, PwLE = People with Lived Experience, RCT = Randomised Control Trial.

^a^Terms used to refer to PwLE and other stakeholders within this table are from the original papers cited here. ^b^Limited information provided.

^c^Lehavot, K., Litz, B., Millard, S. P., Hamilton, A. B., Sadler, A., & Simpson, T. (2017). Study adaptation, design, and methods of a web-based PTSD intervention for women Veterans. *Contemporary Clinical Trials*, *53*, 68-79.
^d^Terp, M., Laursen, B. S., Jørgensen, R., Mainz, J., & Bjørnes, C. D. (2016). A room for design: through participatory design young adults with schizophrenia become strong collaborators. *International journal of mental health nursing*, *25*(6), 496-506.
^e^Terp, M., Bjørnes, C. D., Jørgensen, R., Mainz, J., & Laursen, B. S. (2017). Collaborating with young adults diagnosed with schizophrenia: a participatory design study to shape the healthcare system. *Open Journal of Nursing*, *7*(7), 743-758.

**Supplementary Table 5.** Overview of the reported benefits, challenges and impacts of activities engaging people with lived experience (PwLE)

| **Study details** | **Reported benefits or positive impacts of engagement** | **Reported challenges or negative impacts of engagement** | **Reported changes/decisions made to DMHI as a result of engagement** | **Reported impacts of engagement on DMHI outcomes** |
| --- | --- | --- | --- | --- |
| Abraham *et al*. (2018), USA | - Feedback indicated an increase in identification with CALM content For Veterans Affairs patients’^a^ Authors argue the feedback from the pilot demonstration indicated the adaptions increased relevance and acceptability and thus engagement with the CALM content for patient and stakeholders | - Difficulty retaining focus group participation throughout the study meant not all participants provided feedback across each stage | - Changes to the appearance of the modules - Reducing text on slides - Inclusion of links to videos and other resources - Modifying case studies to better reflect rural veteran experiences | - N/A not reported |
| Batchelor *et al.* (2022), UK | - N/A not reported | - Underrepresentation of participants with low use of COPe-support and those from ethnic minority backgrounds | - Participant reflections in post development stage informed potential future directions and improvements (i.e.. Enhanced visuals, improving navigation, extending access duration). | - Carer feedback was positive overall and indicated the intervention:   - was seen as beneficial  - provided access to credible information  - encouraged personal development and a focus on carer’s wellbeing   - Some carers were prompted to seek additional support |
| Behr *et al.* (2024), Germany | - N/A not reported | - The thoughts and opinions discussed in the focus groups were not necessarily those of all participants - Opposing views between psychotherapists and PwLE meant researchers then had to decide which opinion to include | - Feedback from PwLE and psychotherapists informed the development of the TONI prototype^b^ - Feedback on the prototype resulted in changes to content, language and design - Ability to individualise the intervention - Simple text with inclusive language - Use of a range of media - Inclusive of people with a diverse range of characteristics and disabilities | - High ratings for usability and practicability (SUS score of 83.3 out of a total score of 100 points) - Texts were rated as easy to understand - Participants reported a good fit with outpatient psychotherapy - RCT also in progress (Schaeuffele et al., 2022)^c^ |
| Ben-Zeev *et al.* (2013), USA | - Authors argued the iterative usability testing allowed for the identification of enhancement needs, flaws in the design or content and potential challenges | - N/A not reported | - **Stage 1:** decision by research team to develop a new mHealth resource rather than adapt an existing one - Iterative usability testing: identification of needs and challenges as they arose, resolution of flaws - **Stage 2:** Not reported - **Stage 3:** Removal of abbreviations, reduction and simplification of text, changes to formatting, increased use of images and visual aids - Participants chose the name (FOCUS) | - Participants reported confidence they could use the system (n=12) - Participants reported the system was helpful and easy to use |
| Bucci *et al.* (2019), UK | - Authors reported that the Expert Reference Group was included to ensure the app was more meaningful with appropriate language and processes for end-users | - N/A not reported | - Expert Reference Group informed design and development of app content and protocols - Service users feedback was incorporated into iterations of the app - Changes in functionality, and design of the app based on validation study and Expert Reference Group feedback - Inclusion of additional elements to the app | - A trial of Actissist showed the app was safe, feasible and acceptable (n=24) |
| Callan *et al.* (2021), USA | - N/A not reported | - The patient sample may not be representative more broadly | - Changes to the app’s content, appearance, navigation and organisation based on feedback from patients and CBT therapists^b^ | - Patients utilised the app as well as routine therapy, and reported the app was easy to use and helpful in completing assignments - Moderate association between app use and reductions in depressive symptoms - High ratings of satisfaction and feasibility: - **Stage 1:** Positive ratings on the After-Scenario Questionnaire and Post-Study Satisfactions and Usability Questionnaire. - **Stage 2:** Moderate usability ratings on the Computer System Usability Questionnaire. Patients overall reported positive feedback in the post study feedback interview |
| Danaher *et al*. (2012) USA/Australia | - Enhanced relevance of content and increased credibility of the program | - The views of the focus group may not be representative more broadly due to smaller size | - The feedback was used to: - inform program development - refine content once developed (language and tone, case story examples, protocol for calls, interface and instructions) - inform key components - Feedback led to the development of a website for the Personal Coaches (to view participant progress) | - The participating mothers reported overall positive attitudes to the using program (based on focus groups) - System usability was positively rated (SUS scores) |
| Flobak *et al.* (2021), Norway | - The content represented the intended participants and aligned with their values and experiences which the authors argue is key to the content being seen as meaningful - Mutual learning between clinicians, participants and film production team | - Challenge of balancing the views of clinicians with the lived experiences of participants | - The research team decided to design short videos as the core content based on participant contributions in initial meetings and workshops - Participants contributed to: - the development of the videos (including contributing to decision about content) - design of the behaviour change techniques | - Participants reported: - they could relate and connect to the videos - the characters were seen as “role models for change” |
| Geerling *et al.* (2022), Netherlands | - N/A not reported | - Difficult to differentiate personal vs group opinion within the focus group discussions - Focus groups may not represent broader population | - Feedback from focus groups informed: - exercises and domains included - design (look, sound, use of wording) - inclusion of app personalisation elements | - App users reported they found it beneficial to perform the exercises (91%) - Participants positively rated the app overall (91.5%) and wanted to use the app for an extended period in the pilot (91.5%), and 65.4% of the exercises were completed in the pilot phase, with patients completing more (101/133, 75.9%) exercises than professionals (72/133, 54.1%). |
| Geraghty *et al.* (2016), UK | - Authors argue participants potentially found the intervention relatable and acceptable due to a person-centred approach and engagement of target users in adapting the content | - N/A not reported | - Engaging target users led to: - changes in tone, language and images used - the development of guiding principles - inclusion of information on how the intervention would work - removal of "obvious statements"/ acknowledgment of well-known ideas | - N/A not reported |
| Guala *et al.* (2023), Denmark | - Participants reported finding the game represented situations they could relate to, which then helped with engagement - Authors attributed this to coproduction process | - N/A not reported | - The team (including patients):   - defined the mechanics and style, and created scenarios/situations for the game  - developed the prototype | - Participants viewed Maze Out as useful and acceptable (based on focus group data) |
| Hidalgo-Mazzei *et al*. (2016), Spain | - N/A not reported | - N/A not reported | - N/A not reported | - SIMPLe intervention was found to be: - Feasible (94% continued to use the app at 1 month; 82% after 2 and (74%) at 3 months) - Satisfactory (rated by 86% of participants) - Useful (rated by 82% of participants) |
| Honary *et al.* (2018), UK | - Authors argue the early engagement in the design and evaluation process can increase usefulness of and trust in the DMHI | - Confidentiality concerns around sharing personal experiences of relatives online and fear relatives may see. - Unreliable internet as barrier to engagement | - **Stage 1:** Development: Creation of a resource directory, the REACT group (a moderated online space for sharing knowledge and emotional peer support), and My Toolbox (where participants can store content) - **Stage 2**: User evaluation led to: some rewording, resizing images, creation of additional short instructional videos | - N/A not reported, RCT in progress (Lobban et al; 2017)^d^ |
| Hughes-Barton *et al.* (2023), Australia | - Authors argue engaging with key stakeholders in the design could result in: - meeting the needs of future users better and appealing more to referrers, increasing the likelihood of use - in an intervention that has greater reach to health professionals and people who might not seek support - Authors report it enabled the identification of unique needs and preferences and provided new insights | - Different perspectives from participants made it challenge to balance varied needs (i.e. bright vs calming colours, inclusion of avatars). | - Participants had input into the name and voted on the logo - **Stage 1**: Changes to the webpage design, (look and additional buttons, additional illustrations), development of a safety planning template, inclusion of an information page, increased diversity of images, links to age-appropriate support websites for people under 18 years, inclusion of lived experience videos; minor changes to written content, inclusion of personal keys contacts - **Stage 2:** Increased diversity of images used, inclusion of additional instructions and changes to the text | - High satisfaction rates (15/16 participants) and number of people (15/19 participants) who would recommend icanactnow. |
| Lal *et al.* (2020), Canada | - Authors report a greater understanding of potential barriers, digital technology and necessary adaptions | - Managing diverse expectations | - Modifications made included the addition of local content and links, an additional resource, removal of content specific to Australia, adaptions to the visual display | - N/A not reported |
| Lederman *et al.* (2019), Australia | - N/A not reported | - N/A not reported | - The feedback from workshops informed the design of the intervention, the development of new content and adaption of content to highlight topics more salient to carers | - The responses of carers affirmed that as well as supporting relatedness, the site also supported self-competence and self-directedness/autonomy |
| Lehavot *et al.* (2021), USA | - Authors argue tailoring the treatment to the women Veterans with PTSD may have led to the high levels of engagement and satisfaction found - Authors argue it may have increased the credibility of the treatment and addressed the barriers unique to the target population | - N/A not reported | - Changes to the content, sequencing, and style, revision to language used, inclusion of demonstrative examples | - Compared with phone monitoring, DESTRESS–WV did not have superior reductions in PTSD symptoms however DESTRESS–WV participants reported higher levels of treatment satisfaction - Treatment completers/those with a higher baseline symptom scores had greater PTSD symptom improvement compared to participants in phone monitoring group at 3 month follow up |
| MacKinnon *et al.* (2022), Canada | - N/A not reported | - N/A not reported | - Based on feedback and ongoing input: Designed an app, created a closed-group online forum and used a group therapy format | - Participants engaged in the program (94.1% watching at least one video; 70.6% attending at least 1 telehealth session) - Participants rated the program as a good source of social support (58.8%) - Participants rated the app as easy to learn (64.7%), and useful (58.8%) with 56.3% reporting they were satisfied. - Compared with treatment as usual, BEAM participants showed greater reductions in secondary outcomes (anxiety and sleep problems) but not the primary outcomes (depression or parenting stress) |
| McClelland *et al.* (2018), UK | - Researchers could capture evidence-based content within the app’s structure - Authors argue that without this approach apps are more likely to fail | - Time consuming and expensive - Participants and researchers required skill, flexibility, expertise and patience | - Feedback informed the mock up of the mobile app and the developmental of a version 1 prototype - Consultation resulted in: - personalisation elements - the use of colour to convey mood - inclusion of mood tracking - a calendar linked to a recovery action plan - a help button with links to personal support - evidence based information | - N/A not reported |
| Midgley *et al.* (2021), UK | - Authors argue materials may be more relevant and engaging due to consultation - Authors suggest this may have resulted in higher engagement and retention | - N/A not reported | - The advisory group informed the cultural adaption of the program, in terms of its length, content, use of language and images (incl. logo). | - Reduction in depression and emotion regulation, but not generalised anxiety - Results were maintained at 3 month follow up |
| Milgrom *et al.* (2020), Australia | - See Danaher *et al*. (2012) (above) | - See Danaher *et al*. (2012) (above) | - See Danaher *et al*. (2012) (above) | - Participants engaged well (86% completed all sessions) - Participants reported moderate levels of satisfaction with the program, and high helpfulness ratings - Increased rates of remission from diagnosed depression, and significantly quicker reduction in severity of depressive symptoms - Compared with treatment as usual condition, MumMoodBooster participants had a 4-fold improvement in the rate of depression remission |
| Ospina-Pinillos *et al.* (2019), Australia | - Authors argue cultural preferences could be included and this led to culturally appropriate content and features | - N/A not reported | - Information from workshops informed development of wireframes - Alpha prototype usability testing: - additional of cultural adjustment items in the online self-report assessment, - creation of specific algorithms - development of additional videos and factsheets | - N/A not reported |
| Patterson *et al.* (2022), Canada | - Authors argue the DMHI was more applicable to the real world due to the combination of the expertise of researchers and participants - Improved relevance/usability of the DMHI | - Difficulty scheduling focus groups - Difficulty finding a representative group (small sample (n=6) predominately white, heterosexual women), may limit diversity in perspectives. | - Changes included: - increased personalisation - reduced psychoeducation - increased content accessibility - adaptions to language used - inclusion of ways to tailor the materials to a variety of users - addition of treatment targets - changes to advertisements about Tranquillity - coaches initiate coaching appointments - addition of a screening assessment - additional quality of life and wellbeing tracking | - High rates of usability and likelihood of recommending the DMHI |
| Reupert *et al.* (2020), Australia | - N/A not reported | - N/A not reported | - Inclusion of additional information: managing relationships with peers and others | - mi-spot was found to be safe and acceptable based on participant interview feedback - Significant reductions in participants' depression and stress at 6-week postintervention - Nil online incidents (including online bullying, harassment, violence, or self-harm) - Participants reported feeling safe, cared for, and respected and high satisfaction with the intervention - Participants reported the intervention was easy to follow, assisted their relationships and overall well-being, and was aligned with their values |
| Sin *et al.* (2019), UK | - Engagement of a range of stakeholders - Produced a DHMI that met the requirements of Expert Advisory Group members and carer participants - Usability testing suggests feasible | - Carer sample was limited in in terms of its size and representativeness | - Expert Advisory Group: - Designed and built the intervention including designing study website and online intervention (domain name, content and look) - Integrated feedback from carer consultation - Conducted a walkthrough exercise on beta-build to refine the DMHI. - Carer consultation: feedback sought on alpha build. Based on feedback: the menu design was adapted, elements were colour coded, guidance notes were added, terminology was adapted | - N/A not reported (RCT in progress) ^e^ |
| Terp *et al.* (2018), Denmark | - N/A not reported in this study (see Terp *et al.* (2016))^f^ | - N/A not reported | - N/A not reported | - Less than half, 35% (27/77), of those invited to use the app utilised it - Participants who used the app for more than a month (with their health care professionals) reported positive impact e.g. keeping up medication, feeling they were “a step ahead of their illness”, ability to get appropriate help, feeling more in control of their illness. - Negative impacts reported included increased fears and worries of restraint, increase in uncertainties about their mental health |
| Torok *et al.* (2022), Australia | - Authors argue:   - Engagement of PwLE ensured the app was aligned to the preferences and needs of end users  - high rates of treatment "completion" (71.5%) suggests codesign improved acceptability and relevance | - Challenges in reaching diverse populations meant the sample biased toward females, LGBTQI persons, and those who had previously received treatment. | - N/A not reported | - Compared to control application, LifeBuoy was found to have superior improvements in suicidal ideation severity, but not secondary mental health outcomes |
| Whiteside *et al.* (2019), USA | - N/A not reported | - N/A not reported | - The website was designed in collaboration with Team Matters Now - Creation of videos: Team Now Matters Now members discussed their lived experience and DBT skills to manage suicidal thoughts | - The site was visited by people experiencing current suicidal thoughts - Evaluations suggest visiting the site was associated with reductions in negative emotions and suicidal ideation |
| Wiberg *et al.* (2022), Sweden | - Authors argue a user-centred design:   - contributed to program improvements  - the content was appropriate to the needs and preferences of end-users  - is a useful method for developing a DMHI | - N/A not reported | - Clarified structure for headings - Redesign of a self-monitoring form - Inclusion of a text summary of each video’s content - Creation of a new video demonstration | - Focus group and ‘Think aloud’ feedback indicated participants’ satisfaction - Usability was rated overall as good (based on SUS scores) - High level of acceptability (mean score on the VAS of 77.6%) and treatment completion (73.2%) - Participation associated with significant symptom reductions in core eating disorder symptomology, functional impairment and depressive symptoms. - Results were maintained at 3 month follow up - Some adverse effects of treatment: increased stress and anxiety |

*Note* CBT = Cognitive Behavioural Therapy, DMHI = Digital Mental Health Intervention, DBT = Dialectical Behavioural Therapy, PwLE = People with Lived Experience, SUS = System Usability Scale, VAS = visual analogue scale
^a^Terms used to refer to PwLE and other stakeholders within this table are from the original papers cited here. ^b^ Unclear which specific changes were made based on consultation with PwLE.
^c^Schaeuffele, C., Homeyer, S., Perea, L., Scharf, L., Schulz, A., Knaevelsrud, C., ... & Boettcher, J. (2022). The unified protocol as an internet-based intervention for emotional disorders: randomized controlled trial. *PloS One*, *17*(7), e0270178. ^d^Lobban, F., Robinson, H., Appelbe, D., Barraclough, J., Bedson, E., Collinge, L., ... & Jones, S. (2017). Protocol for an online randomised controlled trial to evaluate the clinical and cost-effectiveness of a peer-supported self-management intervention for relatives of people with psychosis or bipolar disorder: relatives education and coping toolkit (REACT). *BMJ Open*, *7*(7), e016965.
^e^ISRCTN Registry. 2018. Randomised Controlled Trial of COPe-Support Online Resource for Carers URL: <http://www>. isrctn.com/ISRCTN89563420 [accessed 2019-02-12]
^f^Terp, M., Laursen, B. S., Jørgensen, R., Mainz, J., & Bjørnes, C. D. (2016). A room for design: through participatory design young adults with schizophrenia become strong collaborators. *International Journal Of Mental Health Nursing*, *25*(6), 496-506.
